# Local origin of excitatory–inhibitory tuning equivalence in a cortical network

**DOI:** 10.1038/s41593-024-01588-5

**Published:** 2024-03-15

**Authors:** Adrian J. Duszkiewicz, Pierre Orhan, Sofia Skromne Carrasco, Eleanor H. Brown, Eliott Owczarek, Gilberto R. Vite, Emma R. Wood, Adrien Peyrache

**Affiliations:** 1 Montreal Neurological Institute and Hospital, McGill University, Montreal, Quebec Canada; 2 Centre for Discovery Brain Sciences, University of Edinburgh, Edinburgh, UK; 3 Department of Psychology, University of Stirling, Stirling, UK; 4 Ecole normale supérieure, PSL University, CNRS, Paris, France; 5 Simons Initiative for the Developing Brain, University of Edinburgh, Edinburgh, UK

**Keywords:** Neural circuits, Neural encoding

## Abstract

The interplay between excitation and inhibition determines the fidelity of cortical representations. The receptive fields of excitatory neurons are often finely tuned to encoded features, but the principles governing the tuning of inhibitory neurons remain elusive. In this study, we recorded populations of neurons in the mouse postsubiculum (PoSub), where the majority of excitatory neurons are head-direction (HD) cells. We show that the tuning of fast-spiking (FS) cells, the largest class of cortical inhibitory neurons, was broad and frequently radially symmetrical. By decomposing tuning curves using the Fourier transform, we identified an equivalence in tuning between PoSub-FS and PoSub-HD cell populations. Furthermore, recordings, optogenetic manipulations of upstream thalamic populations and computational modeling provide evidence that the tuning of PoSub-FS cells has a local origin. These findings support the notion that the equivalence of neuronal tuning between excitatory and inhibitory cell populations is an intrinsic property of local cortical networks.

## Abstract (web summary)

Cortical excitatory neurons are narrowly tuned to sensory inputs, but the tuning of interneurons is perceived as broad and irregular. Duszkiewicz et al. demonstrate that interneuron tuning is structured and reflects the sum of local excitatory inputs.

## Main

The nature of neural computation is traditionally investigated by determining how external and internal signals are represented at the neuronal level^1–4^. Although neurons in many sensory and other cortical systems encode high-dimensional features^5–8^, their tuning can only be measured for a limited fraction of the possible feature space. In comparison, the feature space of the head-direction (HD) system is relatively simple, with excitatory neurons firing for specific directions of the head in the horizontal plane^4,9,10^. Importantly, this simplicity allows for a full characterization of neuronal tuning during natural behaviors^11,12^.

Cortical inhibition has a critical role in shaping the tuning of neuronal responses^13–21^. Yet, the tuning of inhibitory neurons, especially fast-spiking (FS) cells, is often considered to be broad^22–28^ or irregular^29–33^, begging the question of the origin of such tuning and, specifically, the structure of the underlying circuits. The HD signal is transmitted from the anterodorsal nucleus (ADN) of the thalamus to the cortical recipient neurons in the postsubiculum (PoSub)^9,10,34,35^. Here we recorded the activity of neuronal ensembles in PoSub and ADN and we show that the tuning of PoSub-FS cells is inherited from local but not upstream excitatory cells—an observation further supported by selective optogenetic disinhibition of ADN-HD cells, which modulated PoSub-FS cell activity irrespective of HD. Finally, computational modeling suggests that the distribution of PoSub-FS cell tuning shapes can be accounted for by random and strongly skewed inputs from local PoSub-HD cells.

## Results

### High-density recordings in the PoSub of freely moving mice

**Fig. 1:**
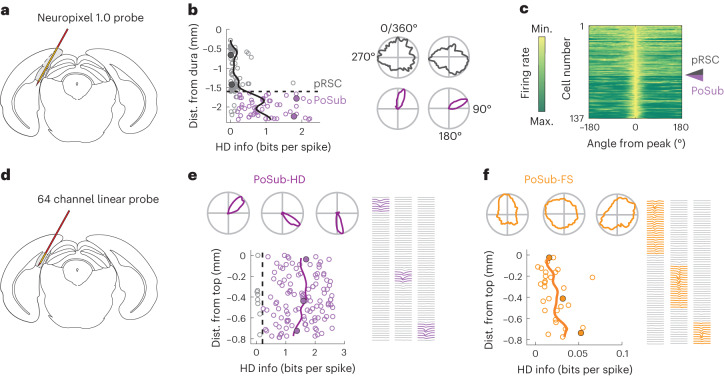
Large-scale recording of neurons in PoSub. **a**, Brain diagram showing the Neuropixel probe implanted along pRSC and PoSub. **b**, Scatterplot depicting HD information of all putative excitatory cells in a single Neuropixel recording as well as the running average (continuous line). Representative HD tuning curves correspond to filled circles. Dashed line shows the putative boundary between pRSC and PoSub. **c**, Normalized tuning curves of all cells in **b**, sorted according to the position on the probe. Arrowhead shows the putative pRSC/PoSub boundary. **d**, Brain diagram showing the 64 channel linear probe implanted parallel to PoSub layers in a subset of animals. **e**,**f**, HD information of all PoSub-HD cells (**e**) and PoSub-FS cells (**f**) in a single recording with a 64 channel probe positioned parallel to cell layers. Solid lines show the running average. Representative HD tuning curves and waveforms (left to right) correspond to filled circles (top to bottom).

**Extended Data Fig. 1:**
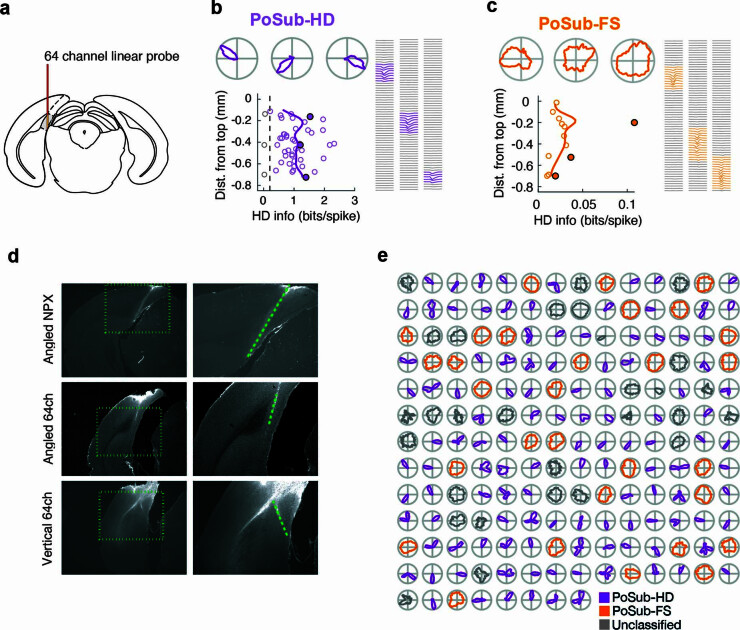
Vertical recordings, examples of probe trajectories, and examples of tuning curves. (**a**) Brain diagram showing the vertical positioning of the 64channel linear probe in PoSub in a subset of animals. (**b**,**c**) HD information of all putative excitatory cells (**b**) and putative FS cells (**c**) in a single vertical 64channel probe recording as well as the running average (solid lines). Representative HD tuning curves and spike waveforms correspond to filled circles. (**d**) Anti-mouse antibody-stained coronal sections depicting the probe tract for the Neuropixel (NPX) recording (top) as well as representative tracts for the angled (middle) and vertical recordings (bottom). Images on the right are magnified regions denoted with a dotted square on the left. Dashed line, position of the probe recording sites. (**e**) Tuning curves of all cells recorded in the example session depicted in Fig. 1d,e, arranged in anatomical order from the top to the bottom of the probe.

**Extended Data Fig. 2:**
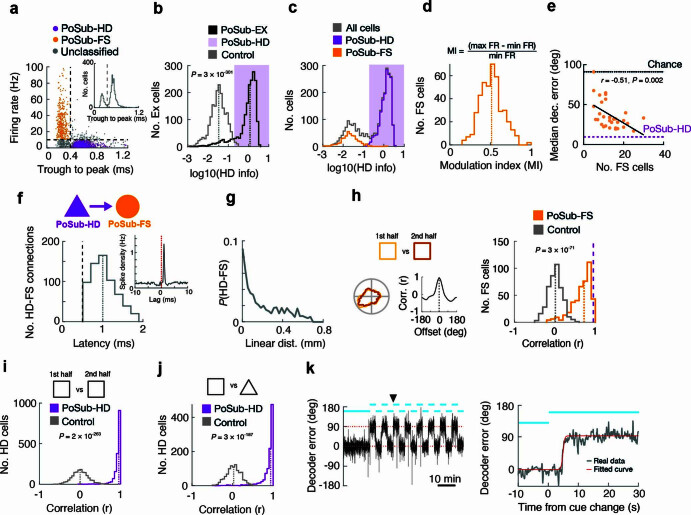
Cell types, synaptic connections, stability, and cue rotation. (**a**) Classification of PoSub units into putative excitatory and putative FS cells. Inset: distribution of trough-to-peak duration among recorded PoSub units. (**b**) Distribution of HD information carried by tuning curves of putative excitatory cells in PoSub (*n* = 1835; Wilcoxon signed rank test vs time-reversed control, *z* = 37.1). Purple shading, PoSub-HD cells. Dotted lines, medians. (**c**) Bimodal distribution of HD information among all recorded PoSub cells. (**d**) Distribution of modulation index of PoSub-FS cell tuning curves. Dotted line, median. (**e**) Median Bayesian decoding error as a function of the number of PoSub-FS cells used. Black dotted line, chance level; purple dashed line, median HD decoding error using PoSub-HD cells (average across all recording sessions). Pearson correlation; *n* = 32 recording sessions. (**f**) Distribution of putative synaptic connection latencies between PoSub-HD cells and PoSub-FS cells. Dashed line, latency threshold. Inset: example spike-timing cross-correlogram between a PoSub-HD and a PoSub-FS cell showing a putative synaptic connection. (**g**) Probability of a putative excitatory synaptic connection between a PoSub-HD and PoSub-FS cell as a function of linear distance on the electrode array. (**h**) PoSub-FS cell tuning curve correlation across two halves of the square arena epoch. Left, example tuning curves of a single PoSub-FS cell and their cross-correlation. Dotted lines, maximum correlation. Right, distribution of correlations (*n* = 427, Wilcoxon signed rank test vs time-reversed control, *z* = 17.8). Dotted lines, medians; dashed line, median of the PoSub-HD cell distribution. (**i**) Distribution of correlations between PoSub-HD cell tuning curves obtained from two halves of the square arena epoch (*n* = 1602; Wilcoxon signed rank test vs time-reversed control, *z* = 34.7). Dotted lines, medians. (**j**) Distribution of correlations between PoSub-HD cell tuning curves in square and triangle arenas (*n* = 1013; Wilcoxon signed rank test vs time-reversed control, *z* = 27.6). Dotted lines, medians. (**k**) Bayesian decoder error during a representative cue rotation session. Left, rotation of the cue by 90 degrees (blue bars) corresponds to the equivalent change in HD decoding error. Right, a single cue rotation epoch (denoted with a black arrowhead on the left). Red line, sigmoidal fit.

We first established the functional border between PoSub and posterior retrosplenial cortex (pRSC) using a Neuropixel linear electrode array (Fig. 1a–c). Based on these observations, a similar step-like increase in average HD tuning along this axis was used to define this border for a larger cohort of mice implanted with a microdrive-mounted 64 channel linear electrode array and record single-unit activity in PoSub. The probe was implanted either vertically (*n* = 931 units from 14 mice, range: 46–101 units per recording; Extended Data Fig. 1a–c) or parallel to PoSub cell layers (*n* = 1,999 units from 18 mice, range: 42–185 units per recording; Fig. 1d,e and Extended Data Fig. 1d,e) and probe positions were later confirmed histologically (Extended Data Fig. 1d). The two datasets were pooled for further analysis. All recording sessions consisted of square open-field exploration and sleep epochs, with a subset of sessions extended to include a triangular open field or a cue rotation task. PoSub units were subdivided into putative excitatory cells (*n* = 1,835) and putative PoSub-FS cells (*n* = 427) based on mean firing rate and waveform shape (Extended Data Fig. 2a). A subset of excitatory cells whose HD information exceeded the 99th percentile of the time-reversed control distribution (Methods) were classified as PoSub-HD cells (>0.2 bits per spike, *n* = 1602, 87% of excitatory cells; Extended Data Fig. 2b,c).

### Tuning of PoSub-FS cells is stable and anchored to landmarks

**Fig. 2:**
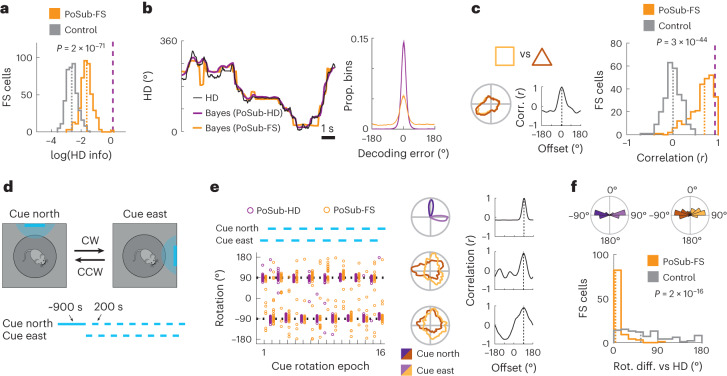
PoSub-FS cells share functional properties with canonical HD cells. **a**, HD information carried by tuning curves of PoSub-FS cells (*n* = 427; Wilcoxon signed-rank test, *z* = 17.9). Dotted lines show medians; dashed lines show the median of PoSub-HD cell distribution. **b**, Bayesian decoding of HD from the activity of PoSub-FS cells. Left, an example period of exploration showing the animal’s true HD and HD decoded from the activity of PoSub-HD cells or PoSub-FS cells. Right, comparison of HD decoding error distributions across all animals (*n* = 32). Shaded area shows s.e.m. **c**, PoSub-FS cell tuning curve correlations across square and triangle arenas. Left, representative tuning curves of a single PoSub-FS cell and their cross-correlation. Dotted lines show the maximum correlation. Right, population histograms (*n* = 264, Wilcoxon signed-rank test versus time-reversed control, *z* = 14.0). Dotted lines show medians; dashed line shows the median of the PoSub-HD cell distribution. **d**, Cue rotation task. Top, diagram of the cue rotation apparatus. Bottom, timeline of the epochs corresponding to different cue positions. **e**, Representative cue rotation session. Left, rotation of PoSub-HD and PoSub-FS tuning curves over 16 consecutive cue rotation epochs (blue lines). Each point denotes the tuning curve rotation of a single cell relative to the previous epoch. Right, representative PoSub-HD and PoSub-FS tuning curves from the same session computed across all CW and CCW epochs and their cross-correlation. Dotted line shows the maximum correlation. **f**, Population data from six cue rotation sessions. Top, distribution of average tuning rotations of PoSub-HD cells (*n* = 411) and PoSub-FS cells (*n* = 99) across all CW and CCW epochs (light and dark shades, respectively). Bottom, histogram of mean absolute rotation differences between individual PoSub-FS cells and the average of PoSub-HD cells (*n* = 99, Wilcoxon signed-rank test versus time-reversed control, *z* = 8.25). Dotted lines show medians. CW, clockwise; CCW, counterclockwise.

We observed that PoSub-FS cells were considerably modulated by HD (Extended Data Fig. 2d) and, at the population level, the quantity of HD information conveyed by PoSub-FS cells was significantly higher than that of the time-reversed control (Fig. 2a). The information contained in PoSub-FS cell tuning curves was sufficient to accurately decode the animal’s HD from PoSub-FS cell spiking activity (Fig. 2b and Extended Data Fig. 2e). However, in contrast to canonical HD cells, individual PoSub-FS cells had complex, often multipeaked tuning curves not confined to a narrow range of HD values. Still, we hypothesized that because PoSub-FS cells receive inputs from local PoSub-HD cells (Extended Data Fig. 2f,g), they should share each other’s functional properties.

Indeed, the tuning of PoSub-FS cells was stable within a single exploration epoch (Extended Data Fig. 2h) and independent of the enclosure geometry (Fig. 2c), reflecting the properties of canonical PoSub-HD cells (Extended Data Fig. 2i,j). Notably, in a cue rotation paradigm^36,37^ (Fig. 2d and Extended Data Fig. 2k), PoSub-FS cell tuning curves followed the rotated distal landmark in concert with PoSub-HD cells (Fig. 2e,f). Thus, although PoSub-FS cells, in contrast to canonical HD cells, exhibit irregular HD tuning curves, their HD tuning is stable and anchored to the landmarks, irrespective of environmental geometry.

### Equivalence of PoSub-HD and FS cell tuning in Fourier space

**Fig. 3:**
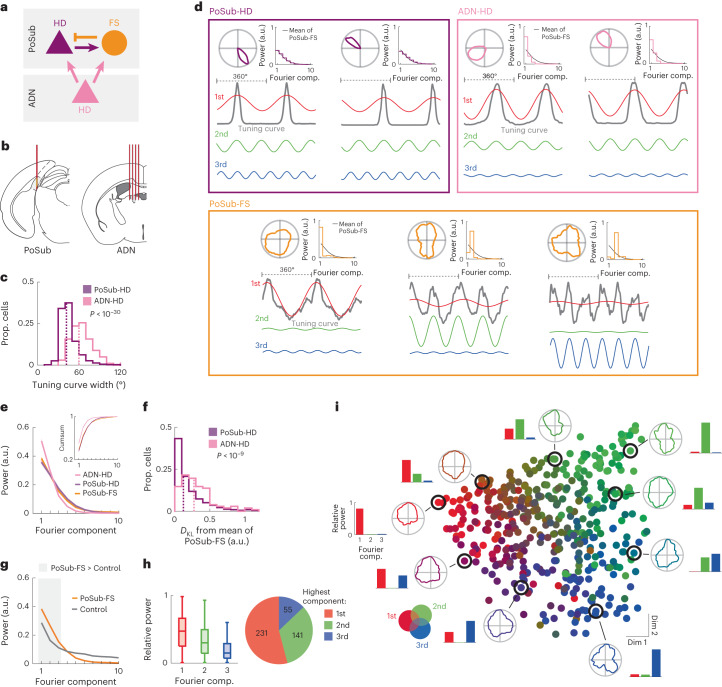
HD tuning of PoSub-HD and PoSub-FS cell populations is equivalent in the Fourier space. **a**, Simplified diagram of connections in the thalamocortical HD system. Arrows and bars show excitatory and inhibitory connections, respectively. **b**, Brain diagrams showing probes in PoSub and the anterior thalamus. **c**, Distributions of tuning curve widths of PoSub-HD and ADN-HD cells (*n* = (1,602, 97), Mann–Whitney *U* test, *z* = 11.4). Dotted lines show medians. **d**, Fourier decomposition of representative PoSub-HD, ADN-HD and PoSub-FS tuning curves. Each example depicts the tuning curve (top-left), normalized power of the first ten Fourier components (top-right) and linearized tuning curve along with the first three Fourier components represented as sine waves (bottom). Black curves show the average PoSub-FS spectrum. **e**, Average Fourier signatures of PoSub-FS, PoSub-HD and ADN-HD cell populations (two-way ANOVA, Fourier component by cell type interaction: *F*_(9,19116)_ = 2.76, *P* = 0.003). Inset, cumulative distribution. Shaded lines show s.e.m. **f**, Statistical distance between individual PoSub-HD cell or ADN-HD cell Fourier spectra and the average Fourier signature of the PoSub-FS cell population (*n* = (1,602, 97), Mann–Whitney *U* test, *P* < 10^−9^). Dotted lines show medians. **g**, Average Fourier signature of PoSub-FS cells compared with time-reversed control (*n* = 427 cells, two-way ANOVA, Fourier component by cell type interaction: *F*_(9,3834)_ = 64.1, *P* = 4 × 10^−110^). Gray background indicates the components for which PoSub-FS group is significantly higher than the control group (Wilcoxon signed-rank test with Bonferroni correction, first: *z* = 0.65, *P* = 8 × 10^−10^; second: *z* = 0.85, *P* = 3 × 10^−16^; third: *z* = 0.46, *P* = 4 × 10^−5^). Shaded lines show s.e.m. **h**, Left, relative mean power of the first three Fourier components for PoSub-FS cells (*n* = 427 cells). Boxes, median and IQR; whiskers, minimum/maximum values that are not outliers. Outliers (>1.5× IQR away from IQR) are not shown. Right, proportions of PoSub-FS cells with the highest power in each of the first three Fourier components. **i**, Isomap projection of PoSub-FS cell tuning curve auto-correlograms, colored using the RGB color model mapped to the relative power of the first three Fourier components. Perimeter, representative PoSub-FS cell tuning curves and the relative power of the first three Fourier components. *D*_KL_, Kullback–Leibler divergence; IQR, interquartile range; RGB, red–green–blue.

**Extended Data Fig. 3:**
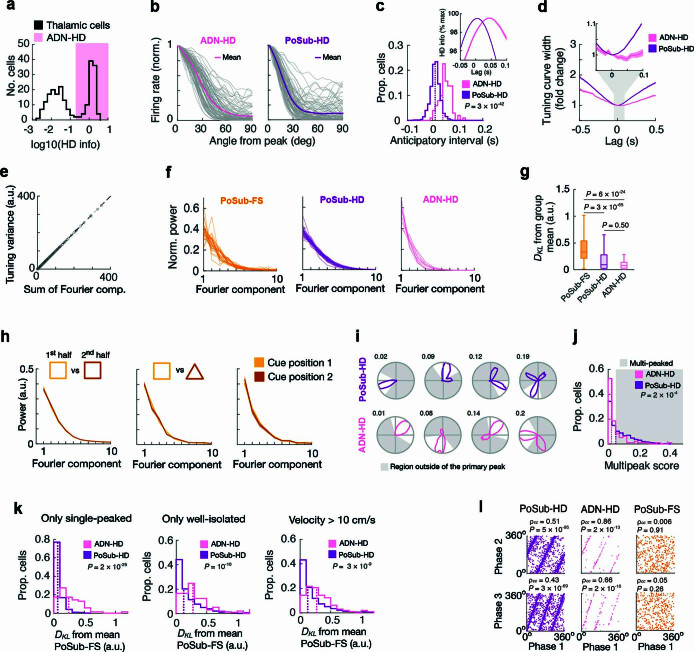
ADN-HD cells, anticipatory intervals, and additional analyses of Fourier signatures. (**a**) Bimodal distribution of HD information in the anterior thalamus. (**b**) Tuning curve slopes of ADN-HD cells (*n* = 97) and PoSub-HD cells (*n* = 97, randomly selected). (**c**) Distribution of anticipatory intervals of PoSub-HD and ADN-HD cells (Mann-Whitney *U*, *n* = (1602, 97), *z* = 13.6). Dotted lines, medians. Inset: HD information as a function of time lag. Shaded lines, mean +/− s.e.m. (**d**) PoSub-HD and ADN-HD cell tuning curve width as a function of time lag. Shaded lines, mean +/− s.e.m. (**e**) Relationship between the sum of Fourier components and tuning curve variance. (**f**) Average Fourier spectra of PoSub-FS, PoSub-HD and ADN-HD cells (individual mice). (**g**) Statistical distance between individual Fourier spectra and corresponding population averages (cell type, *n* = (427, 1602, 97 cells), *F*_(2,2123)_ = 169, P = 5 × 10^−69^; Mann-Whitney *U* (Bonferroni correction): PoSub-FS vs PoSub-HD, *z* = 5.71; PoSub-FS vs ADN-HD, *z* = 3.40; PoSub-HD vs ADN-HD, *z* = 0.45). Boxes, median and the interquartile range (IQR); whiskers, minimum/maximum values that are not outliers. Outliers (>1.5× IQR away from IQR), are not shown. (**h**) Average Fourier spectra of PoSub-FS cells in experimental conditions shown in Fig. 1 (2-way ANOVA, Condition. Left: *n* = 427 cells, *F*_(1,9)_ = 2 × 10^−13^, *P* = 1.00; middle: *n* = 264 cells, *F*_(1,9)_ = 2 × 10^−14^, *P* = 1.00; right: *n* = 99 cells, *F*_(1,9)_ = 6 × 10^−14^, *P* = 1.00. (**i**) Multipeak scores of example tuning curves. (**j**) Multipeak scores in PoSub-HD and ADN-HD cells (Mann-Whitney *U*, *n* = (1602, 97), *z* = 4.25). Dotted lines, medians. (**k**) Statistical distance between individual PoSub-HD cell or ADN-HD cell Fourier spectra and the average Fourier spectrum of the PoSub-FS cells after exclusion of multi-peaked HD cells (left; Mann-Whitney *U*, *n* = (789, 69), *z* = 10.4), after exclusion of the noisiest unit clusters (middle; *n* = (1472, 83), *z* = 5.91), and with a higher velocity threshold (right; *n* = (1602, 97), *z* = 6.46). Dotted lines, medians. *D*_*KL*_, Kullback-Leibler divergence. (**l**) Circular correlation between phases of Fourier components; *n* = 1602 PoSub-HD, 97 ADN-HD and 427 PoSub-FS cells; ρ_cc_, circular correlation coefficient.

We next aimed to establish whether the tuning of PoSub-FS cells was related to the tuning of local HD cells. To address this question, we compared the HD tuning of PoSub-FS cells with that of PoSub-HD cells as well as with that of the HD cells in the upstream ADN (ADN-HD cells; *n* = 97 cells from eight mice; Fig. 3a,b). In mice, ADN-HD cells tend to have broader HD tuning curves than PoSub-HD cells (Fig. 3c and Extended Data Fig. 3a,b), a property independent of their tendency to fire in anticipation of future HD^38^ (Extended Data Fig. 3c,d). We used these differences in tuning curve shape between the two populations to characterize their relative influence on the tuning of PoSub-FS cells. Notably, HD tuning of PoSub-FS cells cannot be directly compared with that of canonical HD cells due to its irregular, often multipeaked shape. To overcome this, we transformed the HD tuning curves from the spatial domain (HD space) to the spatial frequency domain (Fourier space; Fig. 3d,e; Methods). Each tuning curve was thus represented as a sum of sine waves (Fourier components) whose frequencies are equal to the harmonics of the unit circle, corresponding to periods from 360° (fundamental frequency) to 2° (highest possible harmonic, equal to twice the tuning curve sampling bin). In turn, each Fourier component could be described in terms of its amplitude (or ‘power’) and phase, which reflects the relative orientation of that component. Each tuning curve was thus associated with an individual ‘Fourier signature’, consisting of the relative powers of its Fourier components. Across the cell population, the Fourier power decayed rapidly as a function of frequency. Hence, for clarity, we focused our analysis on the relative power of the first ten Fourier components that contained, on average, 98% of the total power.

Canonical HD cells, sharply tuned in HD space, showed broad and stereotyped tuning in Fourier space, with power distributed across several Fourier components and each successive component having progressively less power. We found that Fourier signatures of PoSub-HD and ADN-HD cells often differed between the two regions (Fig. 3d, top)—ADN-HD Fourier signatures tended to be skewed toward lower components compared to PoSub-HD cells, reflecting their broader tuning curves.

In contrast, PoSub-FS cells exhibited highly heterogenous Fourier signatures, with many cells broadly tuned in HD space but narrowly tuned in the Fourier space, that is showing high power for only one Fourier component (Fig. 3d, bottom). We hypothesized that although Fourier signatures of individual PoSub-FS cells reflect their heterogeneous tuning shapes, overall, they ought to be constrained by the tuning properties of their main HD inputs. Indeed, we found that the average Fourier signature of the PoSub-FS cell population was often indistinguishable from the Fourier signatures of individual PoSub-HD cells. In contrast, the Fourier signature of ADN-HD cells was different from both PoSub-HD and PoSub-FS cells, with higher power at low frequencies and lower power at higher frequencies (Fig. 3e,f and Extended Data Fig. 3f). This observation indicates that the tuning of ADN-HD cells is composed of fewer Fourier components than PoSub neurons, irrespective of the exact shape of their tuning curves. Notably, the mean Fourier signature of PoSub-FS cells was stable across different environmental manipulations (Extended Data Fig. 3h), reflecting the stability of their tuning in the HD space. Like hippocampal place cells, HD cells can sometimes have multiple receptive fields^32^ (Extended Data Fig. 3i–j) that could affect the average Fourier signature of an HD cell population. However, the similarity between PoSub-HD and PoSub-FS cell Fourier signatures on a population level was preserved when the analysis was limited to HD cells with a single receptive field. Additionally, it was preserved when a threshold was applied to keep only the best isolated unit clusters or when a higher velocity threshold was used to compute tuning curves (Extended Data Fig. 3k). Thus, although HD tuning curves of individual PoSub-HD and PoSub-FS cells appear strikingly different, on a population level they share the same underlying Fourier power spectrum.

The Fourier components did not equally contribute to the tuning of PoSub-FS cells. Compared to controls, the spectrum of PoSub-FS cells showed higher power for the first three Fourier components only (Fig. 3g), suggesting that these three components form the basis of directional tuning in the PoSub. We classified neurons based on their dominant Fourier components, which reflect the primary symmetries in their tuning curves when represented in polar coordinates. Specifically, neurons were categorized according to the Fourier component that exhibited the maximum power. For instance, a prominent second Fourier component signifies neurons firing in response to two opposite directions, indicative of bilateral symmetry in their tuning. The proportions of recorded PoSub-FS cells with the highest relative power in each of the first three Fourier components were similar to the relative mean power for the population (Fig. 3h).

Although they shared the same average spectrum, PoSub-HD and PoSub-FS cell populations differed in two main aspects. First, while the shape of Fourier signatures was largely uniform among HD cells from the same brain region, it was highly variable among PoSub-FS cells (Extended Data Fig. 3g), indicating narrow tuning of PoSub-FS cells in the Fourier space (Fig. 3d). Hence, the Fourier spectrum of the local HD signal was distributed across the PoSub-FS cell population rather than being homogeneously reflected within each individual cell, often resulting in radially symmetrical tuning curves. Second, in individual HD cells, the phases of Fourier components were correlated with each other, as expected for any symmetrical function with a single maximum (Extended Data Fig. 3l, left and middle). In contrast, the Fourier components of individual PoSub-FS cells had random phases relative to each other (Extended Data Fig. 3l, right), explaining the apparent irregularity in the HD tuning of PoSub-FS cells showing power in more than one Fourier component.

**Extended Data Fig. 4:**
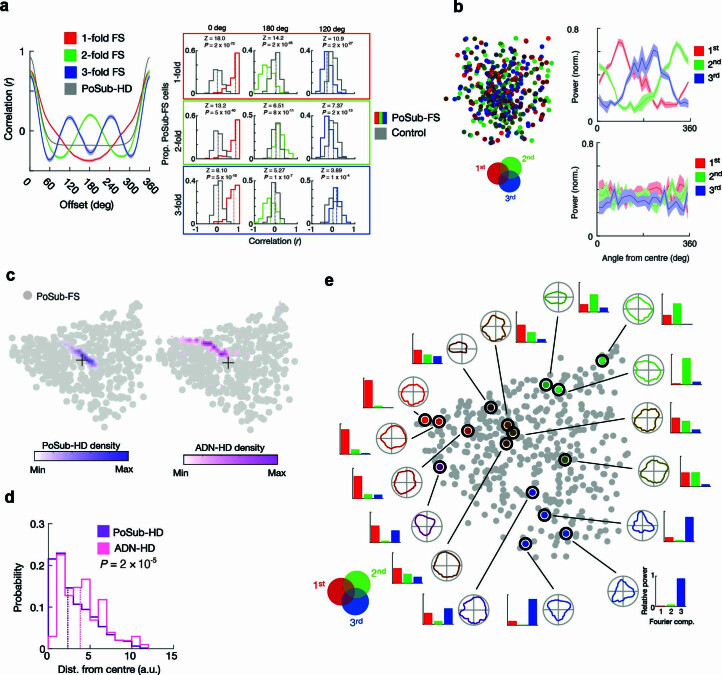
Continuous distribution of the first three Fourier components across the population of PoSub-FS cells. (**a**) Left, average tuning curve auto-correlograms of PoSub-FS cells with the highest power in each of the first three Fourier components, *n* = (231, 141, 55). Note peaks in autocorrelograms at offsets corresponding to different radial symmetries. Shaded lines represent +/− s.e.m. Right, comparisons of the correlation values at the offsets corresponding to radial symmetries for 1-fold, 2-fold and 3-fold PoSub-FS cells with control (time-reversed) distributions (Wilcoxon signed rank test; *n* = (231, 141, 55). (**b**) Left, two-dimensional Isomap projection of time-reversed control PoSub-FS cell tuning curve auto-correlograms. Top right, mean relative power of the first three Fourier components as a function of the angular coordinate from the center of the Isomap projection shown in Fig. 2i. Bottom right, mean relative power of the first three Fourier components as a function of the angular coordinate from the center of the control Isomap projection shown on the left. Shaded lines represent +/− s.e.m. (**c**) Joint two-dimensional projection of PoSub-FS cell tuning curve autocorrelograms (gray points) and (left) PoSub-HD cell or (right) ADN-HD cell auto-correlograms. Values for PoSub-FS cells are shown as individual points (gray) underneath the density distribution of HD cell values. Densities below 0.1 of the maximum value are not shown. Black crosses, centers of the PoSub-FS cell distribution. (**d**) Distribution of Euclidean distances between individual PoSub-HD or ADN-HD cells and the center of PoSub-FS distribution within the Isomap projections shown in **c** (*n* = (1602, 97), Mann-Whitney *U*, *z* = 4.26). Dotted lines, medians. (**e**) Two-dimensional Isomap projection of all PoSub-FS cell tuning curve auto-correlograms (gray points) with all PoSub-FS cells recorded in a single recording session (black circles). Points represent auto-correlograms of individual PoSub-FS cells, colored using the red-green-blue (RGB) color model mapped to the relative power of the first three Fourier components. The projection is surrounded by representative PoSub-FS cell tuning curves coming from a single recording session and the relative power of their first three Fourier components.

To gain further insights into the shape of PoSub-FS cell tuning curves independently of their relative orientation, we computed an auto-correlation function for each cell by correlating its tuning curve with itself at different angular offsets. As expected, cells with maximum power in the first Fourier component showed only one local maximum in the auto-correlograms, at 0° offset (Extended Data Fig. 4a). Auto-correlograms of tuning curves for cells with maximum power for the second component showed a second maximum at 180°, reflecting their twofold radial symmetry. For cells with maximum power in the third component, auto-correlograms peaked at 120° and 240°.

To analyze the shape of PoSub-FS cell tuning curves independently of their Fourier spectrum, we then projected PoSub-FS cell auto-correlograms onto a two-dimensional space using the Isomap dimensionality reduction algorithm^39^ (Fig. 3i and Extended Data Fig. 4b). The resulting projection reflected the heterogeneity of PoSub-FS cell tuning curve shapes across the population (compared to control data; Extended Data Fig. 4b). Notably, the triangular shape of this unsupervised embedding confirmed that a large portion of the power was concentrated in the first three Fourier components. PoSub-FS cells located at each vertex of the triangle showed pure onefold, twofold or threefold symmetrical HD tuning, reflecting their narrow tuning in the Fourier space. Still, this distribution was a continuum as many PoSub-FS cell tuning curves were associated with substantial power in more than one Fourier component (Extended Data Fig. 4e). We then projected the data again into the same two-dimensional space, this time adding the auto-correlograms of PoSub-HD and ADN-HD cells. We found that PoSub-HD and ADN-HD cell tuning curve auto-correlograms occupied compact subspaces within the broader distribution, reflecting their relative homogeneity (Extended Data Fig. 4c). The distribution of PoSub-HD cells occupied the center of the PoSub-FS cell distribution while that of ADN-HD cells was closer to the periphery (Extended Data Fig. 4d), confirming the observations of differences in average Fourier signatures (Fig. 3e,f). In conclusion, the shapes of PoSub-FS cell tuning curves were broadly distributed and each was unique. Yet, the granularity of their tuning was shared with PoSub-HD cells, but not ADN-HD cells.

### PoSub-FS cell tuning reveals key circuit properties

**Fig. 4:**
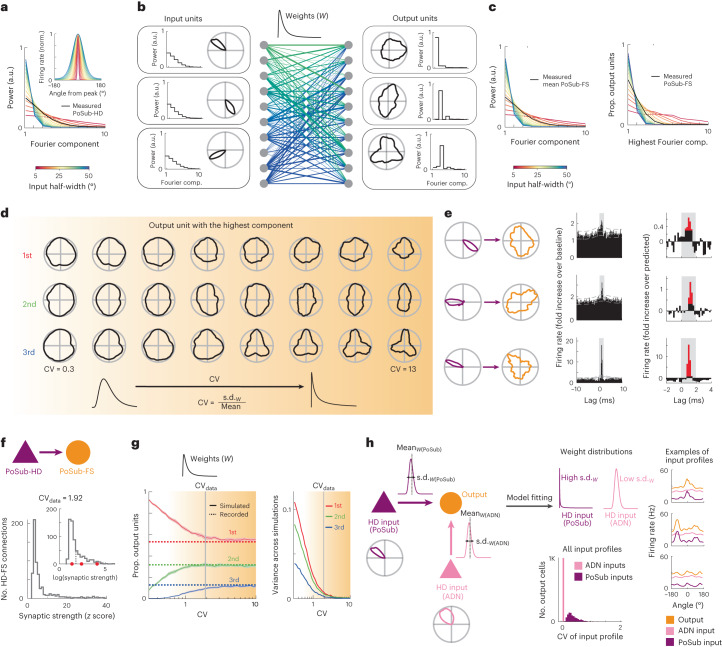
PoSub-FS-like tuning curves emerge from random connectivity in a linear regime and reveal circuit properties. **a**–**c**, Input and output units share the same average Fourier signature in simulation. **a**, Relationship between tuning curve width and Fourier signatures of simulated HD-like input tuning curves. **b**, Emergence of radial symmetries from random feed-forward connections. Left, example input HD tuning curves. Middle, random connectivity with log-normal distribution. Right, output tuning curves resulting from random linear combinations of input HD tuning curves. **c**, Left, mean Fourier power of random linear combinations of input tuning curves. Right, proportions of output tuning curves with highest power in each Fourier component as a function of input tuning curve width. **d**, Output tuning curves with highest Fourier power in the first three components as a function of dispersion of input weights (CV) in an example simulation. **e**, Example tuning curves of putative synaptic PoSub-HD:PoSub-FS cell pairs (left), spike train cross-correlations (middle) and magnifications of putative synaptic peaks (right). Red bars show portions of cross-correlograms significantly higher than predicted. **f**, Distribution of putative synaptic weights in real data (amplitude of the short-latency peak). Inset, same distribution on a logarithmic scale. Red dots show values corresponding to the examples in **f**. Dotted lines show medians. **g**, Left, proportions of simulated cells with maximum power in the first three Fourier components as a function of CV. Dotted lines display the actual proportions of recorded PoSub-FS cells. Shaded area of each curve shows s.d. based on 30 simulations. Right, variance in proportion of simulated cells with maximum power at each component (over 30 simulations). Input weight dispersions exceeding the CV determined experimentally consistently result in proportions close to the observed values. **h**, Fitting the distributions of input weights in a two-input simulation. Left, illustration of the circuit parameters. The model is constrained to match the experimentally determined statistics of the PoSub-FS cell tuning curves. Middle-top, best-fit distributions of the input weights. Right, decomposition of example simulated output cells into the contributions of their ADN and PoSub inputs. Note the flat contribution from ADN compared to the HD-tuned contribution from PoSub. Middle bottom: distribution of CV among contributions from ADN and PoSub. CV, coefficient of variation.

To account for the origin of PoSub-FS cell tuning, we turned to numeric simulations and theory. First, we computed the tuning of output units in a fully connected network receiving HD-tuned inputs (Fig. 4a–c). Output neurons linearly integrated their inputs, as it is the case for cortical FS cells^40^. The spectrum of input tuning curves directly depended on the tuning curve width (Fig. 4a). Output neurons often showed radial symmetries similar to those observed in PoSub-FS neurons (Fig. 4b). Notably, although the input and output tuning curves were strikingly different, random connectivity preserved the Fourier spectrum of the input population (Fig. 4c).

**Extended Data Fig. 5:**
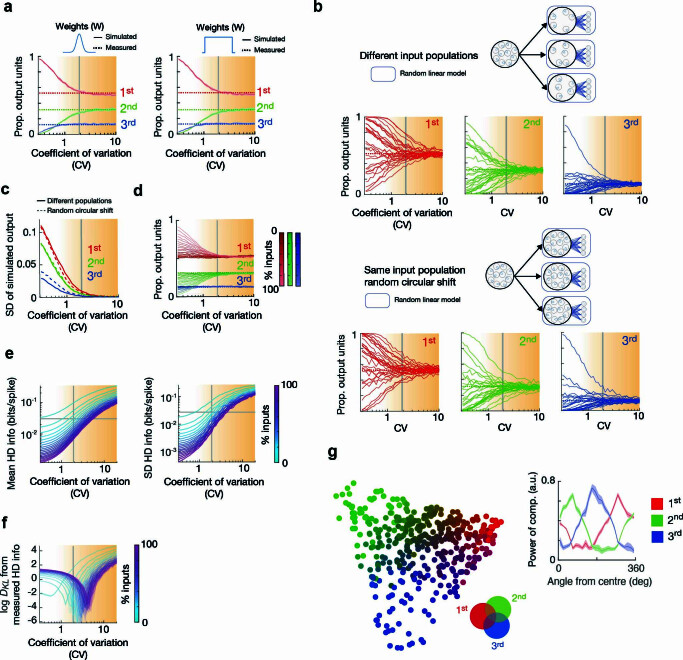
PoSub-FS-like tuning curves emerge from random connectivity in a linear regime. (**a**) Proportion of simulated cells with maximum power in the first three Fourier components as a function of dispersion of the input weight distribution. Left, normal distribution; right, uniform distribution. Dotted lines denote proportions of PoSub-FS cells observed in *in vivo* recordings. Shaded area of each curve, standard deviation (s.d.) based on 40 simulations. (**b**) Proportions of output units with the highest *n*th Fourier component across multiple simulations. Top, sampling from different sub-populations of the pool of input tuning curves. Bottom, sampling from the whole pool of input tuning curves with a random circular shift. (**c**) Quantification of variance across individual simulations shown in **b**. (**d**) Proportion of output units with the highest *n*th Fourier component as a function of connection sparsity (percentage of inputs shared between output units). (**e**) Mean and coefficient of variation (CV) of HD information of simulated output tuning curves as a function of dispersion of input weight distribution and input sparsity. (**f**) Difference in HD information distribution between PoSub-FS cell tuning curves and simulated output tuning curves as a function of dispersion of input weight distribution and input sparsity. *D*_*KL*_, Kullback-Leibler divergence. (**g**) Left, Isomap projection of simulated output tuning curve autocorrelograms. Right, mean relative power of the first three Fourier components as a function of the angular coordinate from the center of the Isomap projection shown on the left. Shaded lines represent +/− s.e.m.

To reveal the conditions under which random connectivity results in output tuning curves with radial symmetries reflecting those of the real PoSub-FS cells, we varied two features of the network: the dispersion of input weights and the number of input units. In simulation, we observed that for low dispersion of the weight distribution, and quite intuitively, all output neurons in the network had similar tuning curves (Fig. 4d). When dispersion of the input weights was increased, individual output tuning curves progressively showed differences in tuning. This was independent of the specific shape of the distribution of weights (Extended Data Fig. 5a). As a result, the proportion of output units showing maximal power in one of the first three Fourier components progressively converged to the proportions observed in PoSub-FS cells (Fig. 4g and Extended Data Fig. 5b–d). This convergence emerged for weight distributions in which the standard deviation (s.d.) exceeded the mean of the weights. To test whether this predicted how PoSub-HD cells were connected to PoSub-FS cells, we quantified the strength of putative synaptic connections from spike train cross-correlations of PoSub-HD:PoSub-FS cell pairs^41^ (Fig. 4e,f; Methods). The distribution was heavy-tailed (Fig. 4f), with the ratio of s.d. and mean input weight (i.e. coefficient of variation), in the range where in silico simulation predicted the emergence of radial symmetries (Fig. 4g).

Varying the parameters of weight distribution did not account for the observed amount of HD information conveyed by PoSub-FS cells (Fig. 2a). Rather, we found that the number of inputs received by each output unit was a key factor influencing the amount of HD information (Extended Data Fig. 5e). Varying both weight distribution and the number of input units, we obtained a distribution of HD information in output tuning curves that matched the real data (Extended Data Fig. 5f), revealing that the tuning of PoSub-FS cells can be used to estimate both the distribution of weights and the number of input neurons. Notably, under optimal network conditions, Isomap projection of output tuning curve auto-correlograms has a similar geometry to that of real PoSub-FS cells (Extended Data Fig. 5g), confirming similar distribution of tuning shapes.

To further quantify the relative contributions of ADN and local PoSub inputs to PoSub-FS cell tuning, we expanded the simulation to include the following two inputs: one with tuning curve widths corresponding to ADN-HD cells and one with tuning curve widths corresponding to PoSub-HD cells (Fig. 4h, left). We then trained the model using gradient descent to find the variances and means of input weights that result in the best fit between the simulated output and real data. The combination of parameters that best described the real data resulted in ADN inputs distributed in a near Gaussian-like manner but a heavy-tailed distribution of PoSub-HD inputs (Fig. 4h, middle). Using these distribution parameters, we performed simulations to determine the contribution of ADN-HD and PoSub-HD inputs to the output tuning curves and established that PoSub-FS cell-like outputs are best explained by flat, high firing rate inputs from ADN-HD cells and low firing rate, HD-modulated inputs from PoSub-HD cells (Fig. 4h, right).

Our simulations, complemented by direct analytical derivation (detailed in the Supplementary Methods), not only support the hypothesis that the symmetries observed in PoSub-FS cell tuning curves originate from local cortical circuits but also demonstrate that these symmetries emerge from strongly skewed distributions of synaptic weights.

### PoSub-FS cells receive directionally uniform thalamic input

Thalamocortical neurons exert a strong excitatory drive onto FS cells in many cortical areas^42,43^, including in the ADN-PoSub circuit^44^. To determine whether upstream thalamic inputs shape PoSub-FS cell tuning, we selectively manipulated the strength (or ‘gain’) of the thalamic input from ADN to PoSub and quantified the effect of this manipulation on the tuning of PoSub-FS cells. We reasoned that if each PoSub-FS cell receives nonuniform thalamic HD input, increasing input gain should result in nonuniform (multiplicative) modulation of their HD tuning. In contrast, if the thalamic input is uniform, PoSub-FS cell tuning should be uniformly (additively) modulated^45^.

**Fig. 5:**
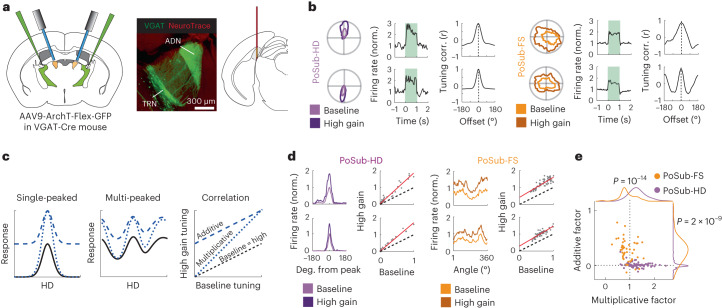
Thalamic drive provides uniform HD input to PoSub-FS cells. **a**, Experimental setup. Left, brain diagram of bilateral AAV injection into TRN and bilateral optic fibers above ADN. Middle, image of a coronal section from the anterior thalamus with extensive inhibitory projections from TRN to ADN. Experiment was repeated four times with similar results. Right, brain diagram of probe placement in PoSub. **b**, Examples of PoSub-HD cell responses (top) and PoSub-FS cell responses (bottom) to the optogenetic increase of thalamic HD gain. Left, HD tuning curves for baseline and high gain epochs. Middle, effect of the optogenetic manipulation on representative cells’ firing rates. Green shading denotes the light pulse. Right, cross-correlation between tuning curves during the baseline and high gain epochs. **c**, Illustration of additive and multiplicative effects of gain modulation on single-peaked (left) and multipeaked tuning curves (middle). Right, correlation between HD tuning curves in baseline conditions (black lines) and high gain conditions (blue lines) reveals the contribution of additive and/or multiplicative factors. **d**, Examples of PoSub-HD cell (left) and PoSub-FS cell (right) HD tuning curves in baseline and high gain epochs plotted in Cartesian coordinates (same examples as in **b**) as well as their respective tuning correlations. Red lines show linear fit. **e**, Values of additive and multiplicative factors for each PoSub-HD and PoSub-FS cell as well as normalized population distributions (*n* = (83, 52), Mann–Whitney *U* test, additive factor: *z* = 7.72, multiplicative factor: *z* = 5.99).

**Extended Data Fig. 6:**
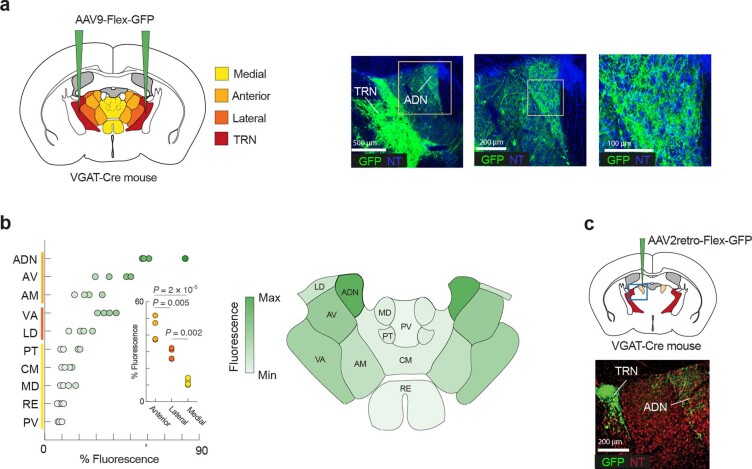
ADN is densely innervated by inhibitory afferents from the TRN. (**a**,**b**), Anterograde tracing of projections from TRN to the rostral thalamus. (**a**) Left, brain diagram showing virus injection sites and sub-divisions of the rostral thalamic nuclei. Right, representative images of fluorescent signal in TRN and neighboring thalamic nuclei. Experiment was repeated 4 times with similar results. (**b**) Left, TRN innervation density in rostral thalamic nuclei (*n* = 4 mice). Inset: density of TRN projections across sub-divisions of the rostral thalamus (repeated measures ANOVA, *F*_(2,9)_ = 44.9, *P* < 10^−4^). Individual comparisons (Bonferroni correction) are displayed on the panel. Data are shown for individual mice. Right, individual rostral thalamic nuclei color-coded according to the TRN projection strength. ADN, anterodorsal nucleus; AV, anteroventral nucleus; AM, anteromedial nucleus; VA, ventral anterior nucleus; LD, laterodorsal nucleus; PT, paratenial nucleus; CM, centromedial nucleus; MD, mediodorsal nucleus; RE, nucleus reuniens; PV, paraventricular nucleus. (**c**) Retrograde tracing of projections from TRN to ADN. Top, coronal diagram showing the virus injection site. Bottom,representative image of fluorescent signal in ADN and anterodorsal TRN. Experiment was repeated 2 times with similar results.

**Extended Data Fig. 7:**
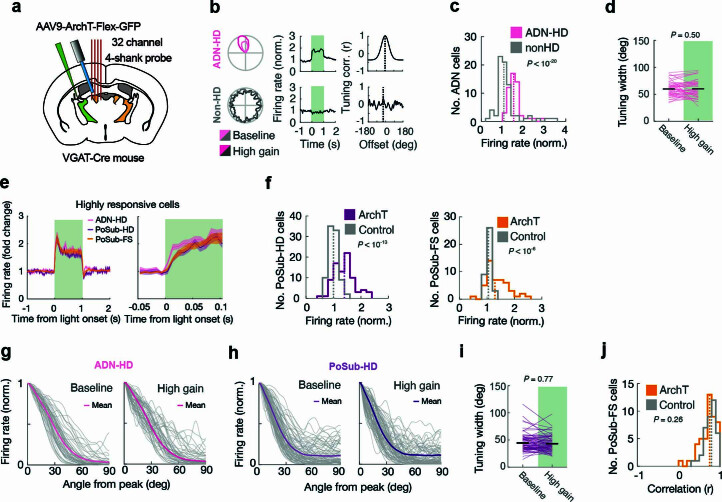
Disinhibition of ADN-HD cells and comparison with other cell populations. (**a**) Brain diagram showing unilateral viral injection into anterior TRN and placement of probe and optic fiber above ADN. (**b**) Examples of ADN-HD cell and thalamic nonHD cell responses to optogenetic inactivation of TRN projections to ADN. Left, HD tuning curves during baseline and high gain epochs. Middle, average effect of the optogenetic manipulation on representative cells’ firing rates. Green shading, light pulse. Right, cross-correlation between HD tuning curves from baseline and high gain epochs. (**c**) Firing rate modulation in ADN-HD and nonHD cells (*n* = (52, 75), Mann-Whitney *U* test between cell types, *z* = 6.57). Dotted lines, medians. (**d**) Width of individual ADN-HD cell tuning curves in baseline and high gain epochs (*n* = 52; Wilcoxon signed rank test, *z* = 0.67, *P* = 0.50). Horizontal lines, medians. (**e**) Temporal profile of ADN-HD, PoSub-HD and PoSub-FS cell responses to optogenetic ADN disinhibition. Only cells with average response above the median for a given population were included (*n* = 26, 41, 23). Shaded areas, +/− s.e.m. (**f**) Firing rate modulation in PoSub-HD cells (left; ArchT, *n* = 83; control: *n* = 89; Mann-Whitney *U* test vs control group, *z* = 1.69) and PoSub-FS cells (right; ArchT, *n* = 47; control: *n* = 38; Mann-Whitney *U* test vs control group, z = 4.99). Dotted lines, medians. (**g**,**h**) Tuning curve slopes of individual ADN-HD cells (*n* = 52) and PoSub-HD cells (*n* = 83) from baseline and high gain epochs. (**i**) Width of individual PoSub-HD cell tuning curves in baseline and high gain epochs (*n* = 52; Wilcoxon signed rank test, *z* = 0.30). Horizontal lines, medians. (**j**) Correlation between PoSub-FS cell tuning curves during baseline and high gain epochs (ArchT group, *n* = 47; control group: *n* = 38; Mann-Whitney *U* test, *z* = 1.14). Dotted lines, medians.

The ADN is strongly innervated by inhibitory afferents from the thalamic reticular nucleus (TRN; Extended Data Fig. 6)^46,47^. We leveraged this specific inhibitory pathway to selectively increase the activity of ADN-HD cells. To that end, we injected a Cre-dependent AAV-ArchT into TRN of VGAT-Cre mice and recorded ensembles of anterior thalamic neurons (Extended Data Fig. 7a; *n* = 127 thalamic cells, including 52 HD cells, from three mice). Targeted illumination of ADN (inactivating the inhibitory presynaptic terminals of the TRN neurons) resulted in a net increase of the firing rates of ADN-HD cells but not of untuned neurons (non-HD cells) recorded in the same sessions (Extended Data Fig. 7b,c), further confirming preferential TRN innervation of ADN over other anterior thalamic nuclei (Extended Data Fig. 6a,b). Contrary to long-term disinhibition^47^, short-term disinhibition of ADN-HD cells was not associated with broadening of their tuning curves (Extended Data Fig. 7d). This manipulation therefore selectively increased the gain of the thalamic HD signal without affecting its granularity. We thus used this method to characterize the effects of thalamic gain modulation on the HD tuning of PoSub neurons. We recorded ensembles of PoSub neurons in VGAT-Cre mice injected with AAV-ArchT (*n* = 83 PoSub-HD cells, 47 PoSub-FS cells from five mice) or a control viral vector (*n* = 89 PoSub-HD cells, 38 PoSub-FS cells from three mice; Fig. 5a) bilaterally into TRN. Similarly to the upstream ADN-HD cells, PoSub-HD and PoSub-FS cells increased their firing rates (Fig. 5b and Extended Data Fig. 7e,f) while preserving their HD tuning (Extended Data Fig. 7g–j).

**Extended Data Fig. 8:**
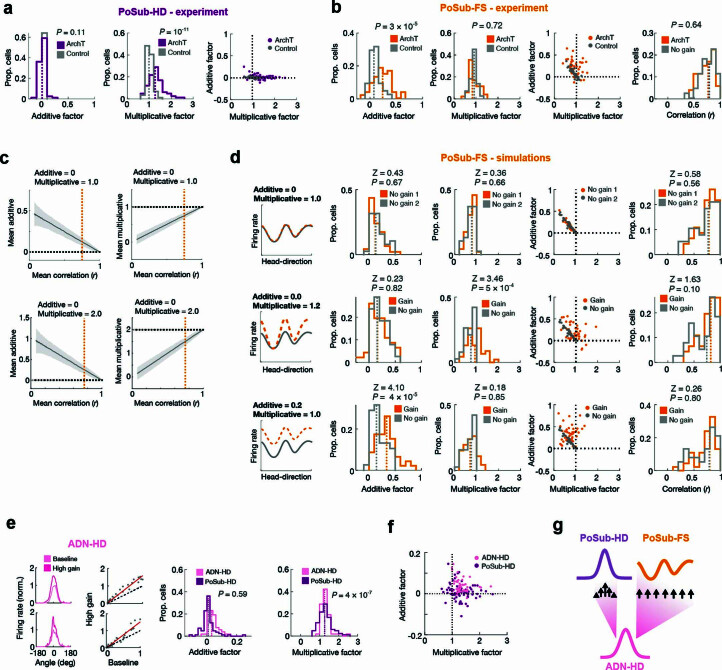
PoSub-HD cells exhibit only multiplicative gain, PoSub-FS cells only additive gain, and ADN-HD cells a mixture of both. (**a**) Left and middle, experimentally determined additive and multiplicative factors for PoSub-HD cells (*n* = (83, 89); additive factor: Mann-Whitney *U* test vs control group, *z* = 1.57; multiplicative factor: Mann-Whitney *U* test vs control group, *z* = 6.80). Dotted lines, medians. Right, relationship between additive and multiplicative factors. (**b**) Left, experimentally determined additive and multiplicative factors for PoSub-FS cells (*n* = (46, 38); additive factor: Mann-Whitney *U* test vs control group, *z* = 4.16; multiplicative factor: Mann-Whitney *U* test vs control group, *z* = 0.36). Dotted lines, medians. Right, contributions of additive and multiplicative factors in PoSub-FS cells and distribution of correlations between unsmoothed baseline and high gain tuning curves (Mann-Whitney *U* test. ArchT vs control group, *z* = 0.46). (**c**) Relationship between correlation of simulated vectors and the apparent values of additive/multiplicative factors. Top, relationship between correlation coefficient and the additive factor (left) or multiplicative factor (right). For low correlations additive and multiplicative factors are over-, and underestimated, respectively. Orange dotted lines, median correlation coefficient between experimentally determined baseline and high gain PoSub-FS cell tuning curves. Black dotted lines, expected magnitudes of additive/multiplicative factors. Shaded lines, +/− 1 s.d. from 1000 iterations. (**d**) Simulations of baseline and high gain tuning curves in noisy conditions. Noise levels were titrated to reflect the experimentally determined distribution of correlation coefficients. Top row, simulations with no gain. Middle row, simulations with multiplicative gain do not reflect the experimentally determined distributions. Bottom row, simulations with additive gain match the experimentally determined distributions. For description of individual panels, see **b**. Statistical comparisons with Mann-Whitney *U* test, *n* = (46, 38). (**e**) Left, examples of ADN-HD cells in baseline and high gain conditions and their respective tuning correlation plots. Red lines, linear fit. Right, comparison of multiplicative factors (Mann-Whitney *U* test, *z* = 0.55) and additive factors (Mann-Whitney *U* test, *z* = 5.07) in ADN-HD and PoSub-HD cell populations. Dotted lines, medians. (**f**) Values of additive and multiplicative factors in PoSub-HD and ADN-HD cells. (**g**) Diagram of the differential effect of thalamic HD input on PoSub-HD and PoSub-FS cell tuning.

We then computed, for each cell, linear regression between HD tuning curves in the baseline condition and under high thalamic gain (that is ADN disinhibition). The slope of the linear fit denotes multiplicative modulation of tuning by thalamic gain, whereas the intercept denotes additive modulation^45^ (Fig. 5c). Thus, a slope above 1 indicates the presence of multiplicative gain and a positive intercept indicates the presence of additive gain. We then assessed the contribution of these additive and multiplicative factors to the tuning modulation of PoSub-HD and PoSub-FS cells. The modulation of PoSub-HD cells was purely multiplicative (Fig. 5d,e and Extended Data Fig. 8a), indicating that they receive HD-specific thalamic inputs. Indeed, this high degree of multiplicative modulation largely reflected the modulation of the upstream ADN-HD cells (Extended Data Fig. 8e,f). In contrast, modulation of PoSub-FS cell tuning was exclusively additive (Fig. 5d,e and Extended Data Fig. 8b). Notably, while uncertainty in tuning curve estimation affects the ability to detect multiplicative gain via linear regression, simulations of gain modulation in the presence of noise point to sufficient detection threshold in our paradigm (Extended Data Fig. 8c,d). Thus, our results indicate that the thalamic inputs received by individual PoSub-FS cells are uniform across all directions (Extended Data Fig. 8g).

### PoSub-FS cells are coupled to the PoSub-HD ring manifold

**Extended Data Fig. 9:**
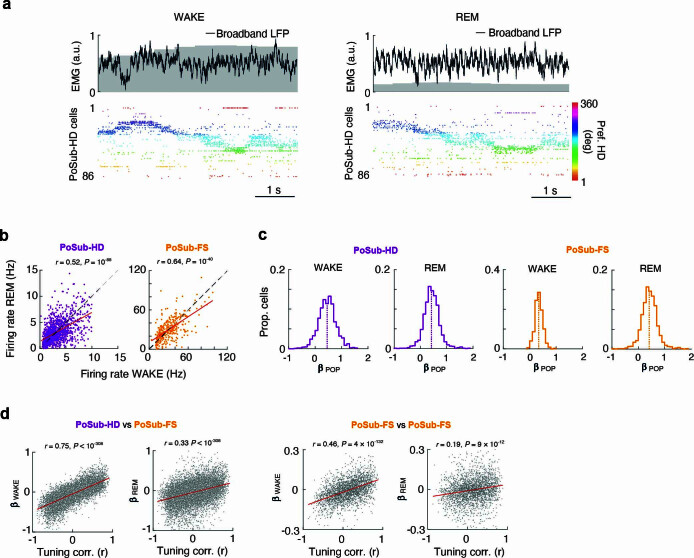
Single unit spiking characteristics, population coupling, and coupling-tuning relationship during WAKE and REM. (**a**) Representative broadband local field potential (LFP) traces and PoSub-HD cell raster plots during WAKE and REM epochs. Gray areas indicate magnitude of LFP-derived electromyogram (EMG, see Methods). HD cells are sorted and color-coded according to their preferred direction during WAKE. (**b**) Relationship between firing rates during WAKE and REM epochs for PoSub-HD and PoSub-FS cells. Pearson correlation, *n* = (1284, 347). (**c**) Distributions of GLM cross-coupling coefficients of individual cells to the population firing rate (β_POP_). (**d**) Relationship between HD tuning correlation and GLM pairwise coupling (β) for PoSub-HD:PoSub-FS and PoSub-FS:PoSub-FS cell pairs during WAKE and REM (Pearson correlation, *n* = 18992 PoSub-HD:PoSub-FS cell pairs, 2492 PoSub-FS:PoSub-FS cell pairs).

Finally, to exclude the possibility that the tuning of PoSub-FS cells is determined by external factors, we sought to establish whether their activity is coupled to the internal attractor dynamics in PoSub in the absence of sensory input. The HD signal in the ADN-PoSub pathway is coherently organized into a one-dimensional (1D) ring attractor even during sleep, when sensory inputs are virtually absent^34,48^. We thus tested whether the tuning of PoSub-FS cells relies on the intrinsic dynamics of the HD cell attractor network during sleep. To address this question, we analyzed ensemble activity during rapid eye movement (REM) sleep, the sleep stage in which coordination of PoSub-HD cells is virtually indistinguishable from wakefulness^34^ (Extended Data Fig. 9a,b).

**Fig. 6:**
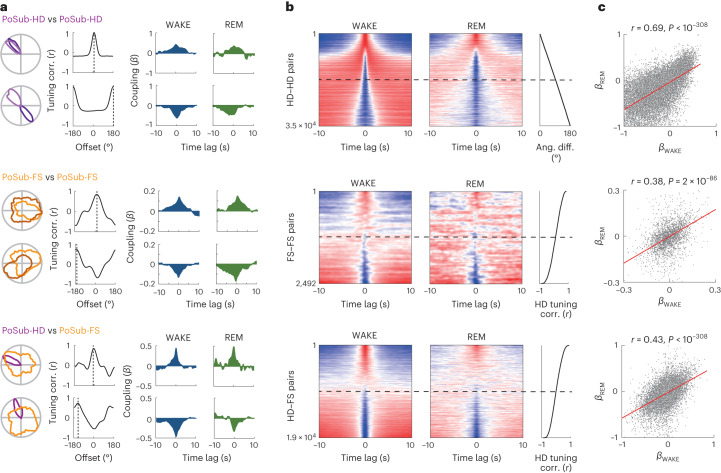
Coupling of PoSub-HD and PoSub-FS cells is conserved across WAKE and REM. **a**–**c**, Cross-coupling relationships between PoSub-HD:PoSub-HD cell pairs (top row), PoSub-FS:PoSub-FS cell pairs (middle row) and PoSub-HD:PoSub-FS cell pairs (bottom row). **a**, HD tuning and spike-timing relationships of representative cell pairs. Left, superimposed HD tuning curves and their HD tuning cross-correlation. Dotted line shows the offset of the maximum correlation. Right, GLM cross-coupling during WAKE and REM. **b**, Color-mapped GLM cross-coupling of all cell pairs during WAKE (left) and REM (middle). Cell pairs were sorted (right) according to the angular difference of their tuning curves (for PoSub-HD:PoSub-HD pairs) or tuning curve correlation at zero offset (PoSub-FS:PoSub-FS and PoSub-HD:PoSub-FS pairs). Each row represents a normalized cross-coupling curve of a single cell pair, color-mapped from minimum (blue) to maximum (red). **c**, Scatter plots illustrating the cross-coupling correlation across WAKE and REM. Red line represents the linear fit. *β* represents the cross-coupling coefficient. Pearson correlation; *n* = 34,270 PoSub-HD:PoSub-HD cell pairs, *n* = 2,492 PoSub-FS:PoSub-FS cell pairs and *n* = 8,992 PoSub-HD:PoSub-FS cell pairs.

We first sought to establish whether the temporal coupling between individual PoSub-FS and PoSub-HD cells was preserved during REM sleep. To account for the coupling to the population firing rate irrespective of any specific tuning, we quantified the pairwise coupling between PoSub cells using a general linear model (GLM)^49^. Although both PoSub-HD and PoSub-FS cells showed strong coupling to the population activity (Extended Data Fig. 9c), we found that the polarity of the GLM cross-coupling coefficient between PoSub-HD cell pairs was preserved across wakefulness (WAKE) and REM sleep (Fig. 6a–c, top). For example, PoSub-HD cell pairs co-active during wakefulness showed a high degree of co-activity during REM sleep, while those that were negatively coupled during WAKE were also negatively coupled during REM. Similarly, PoSub-FS cell pairs also preserved their coupling across WAKE and REM, albeit to a smaller degree than PoSub-HD cell pairs (Fig. 6a–c, middle). Notably, the coupling of the two cell populations to each other was also preserved across WAKE and REM (Fig. 6a–c, bottom). Predictably, the polarity of the cross-coupling coefficient depended on the HD tuning relationship within each cell pair during both WAKE and REM (Extended Data Fig. 9d). Overall, these results indicate that the activity of PoSub-FS cells is coupled to the internal attractor dynamics of the HD system.

**Fig. 7:**
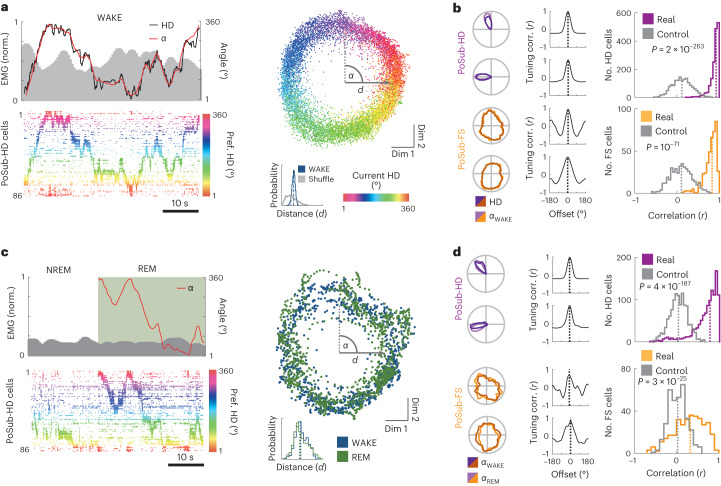
PoSub-FS cells are coupled to the local HD ring manifold during both WAKE and REM. **a**, Isomap projection of HD cell population vectors during WAKE enables unsupervised reconstruction of HD from the ring manifold. Bottom-left, HD cell raster plot of a part of WAKE epoch from a single recording session. Right, Isomap projection of HD population vectors during WAKE. Each point represents a single population vector, color-coded according to the animal’s current HD. Coordinate *α* is the angular position of each population vector on the ring manifold. Distance of population vectors to the center of the manifold (*d*) is indicative of the ring topology. Top-left, coordinate *α* (virtual HD) precisely matches the animal’s current HD. **b**, Left, examples of PoSub-HD and PoSub-FS cell tuning curve reconstructed using the coordinate *α*, and corresponding cross-correlograms between real HD and WAKE Isomap tuning curves. Dotted line shows the maximum correlation. Right, distributions of correlations between real HD and WAKE Isomap tuning curves of PoSub-HD cells (top; *n* = 1,602; Wilcoxon signed-rank test versus time-reversed control, *z* = 34.7) and PoSub-FS cells (bottom; *n* = 427; Wilcoxon signed-rank test versus time-reversed control, *z* = 17.9). **c**, Isomap projection of HD cell population vectors during REM sleep from the same recording session. Green shading shows the REM epoch. Bottom-left, HD cell raster plot of a part of sleep epoch from a single recording session. HD cells are color-coded according to their preferred directions during WAKE. Right, Isomap projection of HD population vectors during REM and WAKE (subsampled). Distance of population vectors to the center of the manifold (*d*) is the same during WAKE and REM. Top-left, coordinate *α* represents virtual HD during REM. NREM sleep epochs were not analyzed. **d**, Left, examples of PoSub-HD and PoSub-FS cell tuning curves reconstructed during REM using the coordinate *α*, and corresponding cross-correlograms between WAKE and REM Isomap tuning curves. Dotted line shows the maximum correlation. Right, distributions of correlation values between WAKE and REM Isomap tuning curves of PoSub-HD cells (top; *n* = 1148; Wilcoxon signed-rank test versus time-reversed control, *z* = 29.2) and PoSub-FS cells (bottom; *n* = 317; Wilcoxon signed-rank test versus time-reversed control, *z* = 10.4). NREM, non-REM.

**Extended Data Fig. 10:**
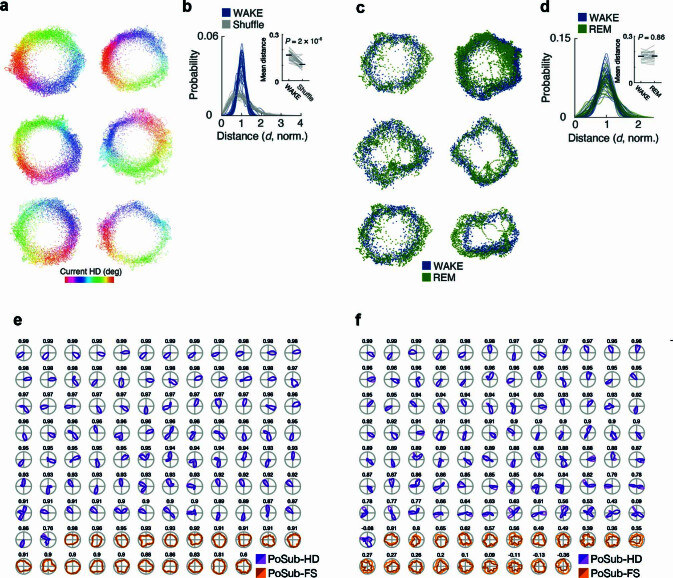
Isomap projections during WAKE and REM and additional tuning curve examples. (**a**) Additional examples of Isomap projections of HD population vectors during WAKE from six recordings. (**b**) Distributions of distances from each point to the center of the manifold for all WAKE Isomap projections of real and shuffled HD population vectors, normalized to the mean distance of the real projections. Each curve represents one recording session. Inset: mean distance to the center of the manifold for real Isomap projections (*n* = 32 mice; Wilcoxon signed rank test vs control, *z* = 4.79). (**c**) Additional examples of Isomap projections of HD population vectors during WAKE and REM from six recordings (same as in **a**). Pearson correlation coefficients are depicted above each pair of tuning curves. (**d**) Distributions of distances to the center of the manifold for all REM and subsampled WAKE Isomap projections, normalized to the mean distance of the WAKE projections. Each curve represents one recording session. Inset: mean distance to the center of the manifold for REM and subsampled WAKE Isomap projections (*n* = 26 mice; Wilcoxon signed rank test, *z* = 0.17). (**e**) Real HD tuning curves (light shades) and WAKE Isomap HD tuning curves (dark shades) of all PoSub-HD and PoSub-FS cells from a single recording (same as in Fig. 6b). Pearson correlation coefficients are depicted above each pair of tuning curves. (**f**) WAKE (light shades) and REM (dark shades) Isomap tuning curves of all PoSub-HD and PoSub-FS cells from a single recording (same as in Fig. 6e).

While stable correlation structures among cell pairs constitute strong evidence for coupling of PoSub-FS cells to the HD attractor network, large-scale population recordings enable a more direct visualization and analysis of the 1D ring attractor manifold that constrains the activity of individual HD cells. We thus asked whether the activity of PoSub-FS cells is constrained by the same ring attractor manifold as that of PoSub-HD cells. To that end, we applied Isomap^39^ to HD cell population vectors to visualize the 1D ring manifold of PoSub-HD cell population activity during WAKE^48,50^ (Fig. 7a and Extended Data Fig. 10a,b). We first confirmed that the internal representation of the animal’s current HD during WAKE can be decoded in an unsupervised manner from the manifold as the angular coordinate (virtual HD) of each HD cell population vector on the ring. The HD tuning curves of both PoSub-HD and PoSub-FS cells computed using virtual HD values during WAKE were equivalent to those computed using real HD values (Fig. 7b and Extended Data Fig. 10e). During REM sleep, the HD system disengages from the outside world while at the same time representing an internally generated, drifting virtual HD^34,48^. We thus applied Isomap to REM PoSub-HD population vectors and computed the corresponding virtual HD (Fig. 7c and Extended Data Fig. 10c,d). As expected, HD tuning curves of PoSub-HD cells generated internally from the animal’s virtual HD during REM were similar to their WAKE counterparts (Fig. 7d and Extended Data Fig. 10f). Notably, HD tuning of PoSub-FS cells could also be accurately estimated during REM based solely on the virtual HD obtained from the HD ring attractor manifold (Fig. 7d). Taken together, these results indicate that the tuning of PoSub-FS cells is restricted by the topology of the HD ring attractor and is largely independent of external inputs.

## Discussion

In summary, our results establish that PoSub-FS cells share many encoding properties with canonical HD cells—their tuning is stable over time and across environments and is anchored to distal landmarks. Remarkably, they often show peaks in their HD tuning curves at intervals corresponding to various radial symmetries, either onefold, twofold or threefold. We further demonstrate the equivalence in the granularity of PoSub-FS and HD cell tuning curves, reflected in virtually identical tuning Fourier spectra of the respective populations. Finally, we found that this relationship is a local property of the network as the tuning of PoSub-FS cells does not depend on the upstream thalamic input from the ADN and is tightly coupled to the intrinsic dynamics of PoSub-HD cells. We predict that this functional relationship between excitatory and inhibitory cell populations is a general feature of cortical neuronal systems.

FS cells integrate and reflect the activity of anatomically proximal excitatory neurons^22,25,33,51,52^. This raises the possibility that despite apparently random connectivity, some PoSub-HD neurons may be organized in local assemblies representing radial symmetries. Previous studies have reported radial symmetries in HD tuning in the retrosplenial cortex^53^ and in spatial tuning in the medial entorhinal cortex in the form of grid cells^3^, border^54^ and band cells^55^, as well as neurons modulated by environment boundaries^56^ and axis of travel^57^ in the subiculum. The retrosplenial cortex, medial entorhinal cortex and subiculum are main output targets of the PoSub. Although HD cell activity in the ADN-PoSub network is crucial for grid cell activity in the medial entorhinal cortex^58^, it remains to be shown whether the organization of PoSub-HD cells into functional and symmetrical assemblies influences downstream spatial symmetries.

The relationship between excitatory and inhibitory tuning that we observed in the cortical HD system may constitute a general principle extending to other cortical systems. Thus, in the primary visual cortex, orientation- and direction-selectivity tuning of excitatory and inhibitory cell populations may show similar equivalence in the Fourier space. What follows is that inhibitory cell tuning may display the same type of radial symmetries observed here but for orientation and direction. Similarly, we predict that in the medial entorhinal cortex, the Fourier signature of grid cell tuning should match the average Fourier signature of the FS cell population within the same module of similarly spaced grid cells. Moreover, FS cells could be tuned to the spatial frequencies of the underlying toroidal topology of grid cell population activity^59^.

## Methods

### Subjects

All procedures were approved by the Animal Care Committee of the Montreal Neurological Institute at McGill University in accordance with Canadian Council on Animal Care guidelines (protocol MNI-7839). The subjects (*n* = 43 for electrophysiology and *n* = 6 for anatomy) were adult (>8 weeks old) male mice bred by crossing wild-type females on C57BL/6J background (Jackson Laboratories, 000664) with either homozygous male VGAT-IRES-Cre mice (Jackson Laboratories, 028862; *n* = 38), PV-IRES-Cre mice (Jackson Laboratories, 017320; *n* = 2) or SST-IRES-Cre mice (Jackson Laboratories, 013044; *n* = 2). One additional mouse implanted with a Neuropixel probe (Fig. 1a–c) was a cross-bred C57BL/6J and FVB (Jackson Laboratory, 001800). Mice were kept in standard conditions (room temperature and 50% humidity) on a 12-h light/12-h dark cycle and were housed in group cages (2–5 mice per cage) before electrode implantation surgery and individually afterwards.

### Electrode implantation

Mice were implanted under isofluorane anesthesia. Silicon probes were mounted on in-house built movable microdrives and implanted through a small craniotomy. Probes were implanted either vertically above left PoSub (from Bregma: AP, −4.24 mm; ML, 2.05 mm; DV, −1.00 mm)^60^ or at a 26° angle pointing away from the midline into left pRSC (from Bregma: anterior-posterior (AP), −4.24 mm; medial-lateral (ML), 1.70 mm; dorsal-ventral (DV), −1.00 mm). A mesh wire cap was then built around the implanted microdrive and was reinforced with UV-cured adhesive. Mice were allowed to recover for at least 1 d before electrophysiological recordings.

Each mouse was implanted with one probe. The probes were either a Neuropixel 1.0 probe (384 active sites arranged in a dense checkerboard layout, that is two columns, 20 μm between each row), a single shank with 64 recording sites (H5; Cambridge NeuroTech) or four shanks with 8 recording sites each (Buzsaki32; NeuroNexus). In all experiments, both ground and reference wires were soldered to a single 100 μm silver wire, which was then implanted above the cerebellum.

### Behavioral procedures

Before the implant surgery, mice were habituated over several days to forage for small pieces of Honey Cheerios cereal (Nestle) in the open field. For most recordings, the recording chamber consisted of a metal frame (90 × 90 × 180 cm) supporting a plastic platform with removable walls (width, 80 cm; height, 50 cm) that could be arranged into either a square or triangular open field. Recordings of two mice implanted in ADN were conducted in a circular open field (diameter, 85 cm; height, 50 cm). The recording protocol consisted of a sleep session in the home cage, followed by open-field exploration in a square arena and another sleep session. A subset of animals then explored a triangular arena. A white rectangular cue card on one of the walls served as a salient landmark.

### Recording procedures

During the recording sessions with 32- or 64-channel silicon probes, neurophysiological signals were acquired continuously at 20 kHz on a 256-channel RHD USB interface board (Intan Technologies) and captured with Intan RHX software (Intan Technologies). For the Neuropixel recording, neurophysiological signals were acquired continuously at 20 kHz on a control board (Native Instruments) and captured with SpikeGLX 3.0 (https://billkarsh.github.io/SpikeGLX/). The wide-band signal was downsampled to 1.25 kHz and used as a local field potential(LFP) signal. The recording cables were tethered to a motorized electrical rotary joint (AERJ; Doric Lenses). Ahead of the main recording session, the microdrive was lowered over several hours in small (35–70 μm) increments until the whole shank was positioned in PoSub or ADN. A short open-field session was then recorded to map the HD receptive fields of all neurons. For PoSub, the recording depth was adjusted so that sharply tuned HD cells (a hallmark of PoSub) were present along the whole length of the shank. Data collection did not commence until at least 2 h after the last depth adjustment. For animals implanted with single-shank linear probes, only one session per mouse was included in each analysis to prevent double-counting of cells. For animals implanted with four-shank Buzsaki32 probes, multiple sessions per mouse (obtained on separate days) were included in the analysis, ensuring that the probe was moved by at least 70 μm between the recording sessions.

Animal position and orientation were tracked in 3D using seven infrared cameras (Flex 13; Optitrack) placed above the enclosure and coupled to the Motive 2.0 motion capture system (Optitrack). Seven small tracking markers were attached to the headcap. Video recording was captured by an overhead camera (Flex 13; Optitrack) placed close to the rotary joint. Animal position and head orientation were sampled at 100 Hz and were synchronized with the electrophysiological recording via voltage pulses registered by the RHD USB interface board (Intan Technologies).

### Optogenetic experiments

VGAT-IRES-Cre mice were injected with an adeno-associated virus vector (AAV 2/9 CAG-Flex-ArchT-EGFP or CAG-Flex-EGFP, titer: 4–5 × 10^12^ genomic copies (GC) per ml; Neurophotonics) into TRN (from Bregma: AP, −0.70 mm; ML, 1.25 cm; DV, −3.25 cm) under isofluorane anesthesia either unilaterally for ADN recordings or bilaterally for PoSub recordings. Injections (300–400 nl per injection site) were done with a microinjector (Harvard Apparatus) through a small craniotomy, at the speed of 100 nl s^−1^. The needle was left in place for 2–5 min after injection.

At least 3 weeks after the injection surgery, optic fiber implants (Doric Lenses; MFC_200/240-0.22_25mm_SM3) were implanted unilaterally (left hemisphere, ADN recordings) or bilaterally (PoSub recordings) above ADN at a 20° angle from the sagittal plane (from Bregma: AP, −0.82 mm; ML, 1.00 cm; DV, −2.25 cm). Mice were then implanted with a microdrive-mounted Buzsaki32 probe above left ADN (Bregma: AP, −0.82 mm; ML, 0.85 cm; DV, −2.00 cm) or either Buzsaki32 probe (Neuronexus) or H5 probe (Cambridge Neurotech) above left PoSub, as described above.

Laser light was delivered from a 520 nm fiber-coupled laser diode module (Doric Lenses) controlled with a laser diode module driver (Doric Lenses). Light power output at the tip of the fiber implant was measured before each implantation and an output curve was calculated individually for each implant. Light output was then set to 14–16 mW before each recording session. For this subset of mice, the second sleep session was followed by a second exploration session in the open field, during which a laser stimulation protocol was delivered via patch cords attached to the optic fiber implants. After 5 min of exploration, light pulses (1 s) were delivered at 0.2 Hz in groups of 60 (5 min total), each followed by 5 min of no stimulation. Four such epochs were delivered in total, resulting in 240 light pulses over a 45-min recording session.

### Cue rotation experiment

A subset of animals implanted into PoSub with linear probes underwent a cue rotation experiment in a separate recording session. To this end, the frame of the recording chamber was fitted with black plastic insets that covered the floor (90 × 90 cm) and walls to obstruct any visual cues. Each of the four walls had an identical panel made of two light-emitting diode (LED) strips (yellow V-shape or blue X-shape) in the center. A small (diameter: 30 cm) elevated circular platform was placed in the center of the arena. Before each recording session, two adjacent LED panels were chosen as distal visual cues. The LED light intensity was titrated so that the panels were visible in the dark but provided minimal illumination of the surrounding area. The on/off cycle of the LED panels was controlled with an Arduino microcontroller. To reduce the specificity of olfactory and auditory cues, the whole recording chamber was thoroughly cleaned with antibacterial wipes before the experiment and white noise was emitted from speakers placed underneath the chamber.

The recording protocol consisted of a 1-h sleep session in the home cage followed by a 75-min cue rotation session. At the start of the cue rotation session, a single LED cue was switched on, and the mouse was placed directly on the circular platform and was left undisturbed for the duration of the session. After 15–20 min of exploration with a stable cue, the cue rotation protocol was initiated. The protocol consisted of the illuminated cue switching back and forth between two adjacent walls every 200 s for a total of 16 rotations. Additionally, to habituate the mouse to the cue disappearing from its field of view, the cue was switched off for 0.1 s every 20 s. To encourage the mouse to explore the platform for the whole duration of the session, the experiment was conducted in the middle of the dark phase of the light cycle and the recording chamber was sprayed with a new odor (scented air freshener) right before the cue rotation session.

### Tissue processing and probe recovery

Following the termination of the experiments, animals were deeply anesthetized and perfused transcardially first with 0.9% saline solution followed by 4% paraformaldehyde solution. The microdrive was then retracted to remove the probe from the brain. Brains were sectioned with a freezing microtome coronally in 40 μm slices. Sections were washed, counterstained with DAPI and Green Neurotrace and mounted on glass slides with ProlongGold fluorescence antifade medium. Sections containing probe tracts were additionally incubated with a Cy3 anti-mouse secondary antibody (1:200 dilution; Cedarlane, 715-165-150) to help visualize the electrode tract. Widefield fluorescence microscope (Leica) was used to obtain images of sections and verify the tracks of silicon probe shanks, optic fiber position and virus expression.

### Spike sorting and unit classification

Spike sorting was performed semi-automatically using Kilosort 2.0 (ref. ^61^) followed by manual curation of the waveform clusters using the software Klusters^62^ or Phy^63^. At this stage, any cluster without a clear waveform and clear dip in the spike train auto-correlogram at the 0–1 ms time bin was classified as noise and cluster pairs with similar waveforms and a clear dip in their spike train cross-correlograms at the 0–1 ms time bin were merged.

For PoSub recordings, viable units were first identified as units that (1) had an average firing rate of at least 0.5 Hz during open-field exploration and (2) had a waveform with negative deflection (criterion aiming to exclude spikes from fibers of passage). Next, putative excitatory cells and putative FS interneurons were classified on the basis of their mean firing rate during open-field exploration and the through-to-peak duration of their average waveforms (Extended Data Fig. 2a). Putative FS interneurons were defined as cells with short trough-to-peak duration (<0.4 ms) and high mean firing rates (>10 Hz). Conversely, cells with long trough-to-peak (>0.4 ms) and low mean firing rates (<10 Hz) were classified as putative excitatory cells.

### HD tuning curves and tuning metrics

The animal’s HD was calculated as the horizontal orientation (yaw) of a polygon constructed in Optitrack tracking software by connecting the three-dimensional coordinates of all tracking markers on the animal’s headcap. The yaw of the polygon was measured in the global coordinates (that is, the axes of the environment, not the axes of the polygon), and these were constant across the whole study. HD tuning curves were then computed as the ratio between histograms of spike count and total time spent in each direction in bins of 1° and smoothed with a Gaussian kernel of 3° s.d. Tuning curves were computed from epochs when the animal’s speed exceeded 2 cm s^−1^ for all analyses except cue rotation and optogenetic experiments, where epoch duration (200 and 240 s, respectively) was too short to allow for further refinement.

To prevent any bias in HD cell population due to assumptions about the unipolar shape of HD cell tuning curves, we chose to define HD cells based on HD information contained in the tuning curves^64^, calculated for *n* angular bins as:

$$I=\sum _{i=1.n}\lambda ({\Theta }_{i}){\log }_{2}\left(\frac{\lambda ({\Theta }_{i})}{\lambda }\right)p({\varTheta }_{i})$$

where *λ*(*Θ*_*i*_) is the firing rate of the cell for the *i*th angular bin, *λ* is the average firing rate of the neuron during exploration and *p*(*Θ*_*i*_) is the occupancy (that is, normalized time spent) in direction *Θ*_*i*_. This information rate (measured in bit s^−1^) was normalized by the cell’s average firing rate to provide an information content (measured in bit spike^-1^) independent of firing rate.

For each cell, we obtained the control tuning curve using a time-reversed HD angle—a method that preserves the dynamics of both the spike train and the HD angle but decouples the two from each other. We computed the time-reversed HD angle by reversing the order of HD angle values with respect to their timestamps within a particular epoch. We then classified HD cells as those with HD information scores higher than the 99th percentile of the null distribution (>0.2 bits per spike, 85% of putative excitatory cells). We did not apply any HD information threshold to PoSub-FS cells.

### Cross-validated HD tuning curve auto-correlograms and cross-correlograms

We obtained HD tuning curve auto-correlogram and cross-correlogram by computing Pearson’s correlation coefficients between the reference tuning curve vector and the second tuning curve vector (from either the same or another cell), which was circularly shifted by 0–359 bins. To minimize the effect of non-HD factors on tuning curves computed from the same epoch, we used a cross-validation procedure whereby the two tuning curves were computed from separate halves of the epoch.

When computing HD tuning curves during the exploration of the triangular open field, we noticed that sometimes the cells’ receptive fields were rotated with respect to the prior square open-field exploration. To correct for this, we first calculated for each HD cell the degree of tuning curve rotation between the two environments via cross-correlation. We then used the average rotation per recording session to circularly shift all triangular open-field tuning curves in the opposite direction by the equivalent amount. This allowed us to compute the true tuning curve correlation between the two environments.

### Detection of monosynaptic connections

Spike train cross-correlograms of ±50 ms binned in 0.2-ms windows were convolved with a Gaussian kernel of 4 ms s.d, resulting in a predictor of the baseline rate. At each time bin, the 99th percentile of the cumulative Poisson distribution (at the predicted rate) was used at the statistical threshold for significant detection of outliers from baseline. A putative connection was considered significant when at least two consecutive bins in the cross-correlograms passed the statistical test.

### Analysis of HD cell realignment after cue rotation

To estimate the degree of realignment of the HD system following cue rotation, HD cell spike times were binned into population vectors (50 ms window, smoothed in 100 ms s.d. Gaussian windows). Based on cells’ tuning curves from the baseline period of exploration with a stable cue, the population vectors were converted into a Bayesian probabilistic map under the hypothesis that HD cells fire as a Poisson process. The instantaneous internal HDs were taken as the maxima of these probabilistic maps. These estimates faithfully tracked the head orientation of the mouse during the period preceding cue rotation. The degree of realignment of the HD system was calculated as the decoder error—angular difference between the real HD and the decoded HD at each time bin. Because not all cue rotations resulted in HD realignment, we excluded all cue rotation epochs that (1) resulted in less than 45° of mean decoder error in the following 200 s epoch and (2) occurred when the animal was stationary in the preceding epoch (average velocity <2 cm s^−1^).

To estimate the point at which the HD system remaps, we fitted a sigmoid curve to the decoder error values following each cue rotation using the *sigm_fit* function (https://www.mathworks.com/matlabcentral/fileexchange/42641-sigm_fit). We defined the beginning and end of realignment epochs as the timestamps corresponding to the values of 0.01 and 0.99 of the normalized sigmoids. We then, for each cell, calculated an HD tuning curve for the remainder of each cue epoch (from the end of realignment to the next cue rotation) and computed the cross-correlation (see above) between HD tuning curves from consecutive epochs. For each cell, the degree of realignment was defined as the tuning curve offset that results in the highest correlation coefficient. The difference in realignment between FS and HD cells was defined as, for each FS cell, the angular difference between its degree of realignment and the average realignment of HD cells in the same epoch.

### Classification of sleep states

Sleep scoring was performed using the automated SleepScoreMaster algorithm and TheStateEditor software^65,66^ (Buzsaki Laboratory, https://github.com/buzsakilab). The wide-band signal was downsampled to 1.25 kHz and used as the LFP signal. Electromyograph (EMG) was computed from correlated high-frequency noise across several channels of the linear probe. Recording sessions with less than 100 s of REM sleep detected (*n* = 6) were excluded from the analyses involving REM sleep because of low number of samples.

### Pairwise spike–rate coupling

Quantification of pairwise spike–rate coupling between cells was quantified using a GLM according to the method described in ref. ^49^. Spike trains were binned in 100 ms bins and smoothed in 100 ms s.d. Gaussian windows. The population firing rate was calculated by aggregating all spike times from all recorded units in a given recording and processing them in the same manner as single spike trains. Both binned trains were then restricted to either WAKE or REM epoch (see above).

The GLM was fitted using the MATLAB *glmfit* function. The binned spike train of cell A was modeled as a Poisson process, as a function of both the binned spike train of cell B and the binned population firing rate, using a log link function. The model produced a coefficient of coupling between the spike trains of cells A and B (‘β’), as well as a coefficient for the coupling of cell A to the population firing rate (‘β_POP_’). The procedure was repeated by offsetting the spike train of cell A by ±10 s in 100 ms intervals, to yield the equivalent of the spike train cross-correlogram that discounts the coupling of cell A to the local population rate. Because this procedure, unlike Pearson’s correlation, is not symmetric, it was repeated by swapping cell A and cell B and averaging the coupling coefficient values at equivalent offset intervals. A cell pair was removed from the analysis in rare cases when the *glmfit* function identified the model as ill-fitted or reached the iteration limit.

### Visualization and analysis of the ring manifold

For the visualization of the HD manifold during WAKE, HD cell spike times from the whole epoch were binned into population vectors in 200 ms bins, smoothed in 400 ms s.d. Gaussian windows and a square root of each value was taken. Then, nonlinear dimensionality reduction was performed using the Isometric Feature Mapping (Isomap) algorithm^39^ implemented in the MATLAB Toolbox for Dimensionality Reduction (version 0.8.1b, https://lvdmaaten.github.io/drtoolbox/). The parameters were set to 12 nearest neighbors and three dimensions—the latter to inspect if there is no higher dimensional manifold in the data. Shuffled Isomap embeddings were computed by shifting each cell’s binned spike train in time by a random number of bins.

Internal HD at each time bin was then calculated as a four-quadrant arctangent of the first two Isomap dimensions (range: −180° to 180°). Notably, the ‘virtual HD’ generated this way has arbitrary directionality (clockwise/counterclockwise) and an arbitrary point of origin. To align it, Isomap directionality was established by computing the Isomap error as the difference between real HD values and (1) virtual HD values and (2) additive inverse of virtual HD values, and selecting the directionality for which the distribution of angular differences between real and virtual HD had smaller circular variance. Internal HD tuning curves were then computed as the ratio between histograms of spike count and total time spent in each virtual HD bin (1°) and smoothed with a Gaussian kernel of 3° s.d. Real HD tuning curves were computed by downsampling the real HD into 200 ms bins and applying the same procedure as above. To correct for the arbitrary point of origin, HD tuning curve cross-correlations (see above) were computed between the real and virtual HD tuning curve of each HD cell, and the mean offset of maximum correlation was then used to circularly shift all tuning curve vectors by the equivalent number of bins. Although this procedure (as well as Isomap mapping in general) was dependent on the real HD tuning of HD cells, it was independent of FS cell tuning. Control HD tuning curves were computed by time-reversing the Isomap angle (see above).

For the comparison between Isomap HD tuning curves during WAKE and REM, population vectors across WAKE and REM were computed in the same manner as above. WAKE population vectors were then randomly downsampled to equal in number to the REM population vectors. Isomap algorithm was then run on both WAKE and REM population vectors together. Virtual HD was computed in the same way as above. HD tuning curves were computed in bins of 6° and smoothed with a Gaussian kernel of 6° s.d for both real and internal HD. Larger bin size was chosen due to sometimes uneven occupancy of virtual HD during REM.

### Fourier analysis of HD tuning curves

To decompose tuning curves into Fourier series, we projected the tuning curves onto a basis of sine and cosine functions whose frequencies were the harmonic of the unit circle, that is, from the fundamental frequency (period of 360°) to the highest possible frequency (2°, the inverse of the Nyquist frequency as tuning curves were computed in 1° bins). The power, or Fourier coefficient, at a particular frequency was defined as the root mean square of the projection values onto the sine and cosine basis at that frequency. Similarly, the phase was defined as the arctangent of the projections. The validity of the projection was verified by checking that the sum of squared Fourier coefficients is equal to the variance of the tuning curves (Parseval’s identity), which was indeed the case (Extended Data Fig. 3e). Because higher Fourier components likely represent noise fluctuations in the tuning curves, we focused our analysis on the relative power of the first ten Fourier components, normalizing their individual power values to the sum of their power.

Kullback–Leibler (KL) divergence was used as a measure to assess the similarity between the individual Fourier spectra and the population means. While KL divergence is regularly used to compare probability distributions, we deemed it appropriate to apply it to normalized Fourier spectra as they were mathematically indistinguishable from probability distributions. We thus computed the KL divergence between the spectrum of an individual neuron *σ*_*i*_ (*k*) (with $k{\epsilon }[1.10]$ the angular frequencies) and the average Fourier spectrum of a population *S*_*i*_ (*k*) as follows:

$${D}_{\rm{KL}}({\sigma }_{i}||S)=\sum _{k=1.10}{\sigma }_{i}(k)\log \left(\frac{{\sigma }_{i}(k)}{S(k)}\right)$$

While KL divergence is not symmetrical, that is, KL divergence (*D*_KL_) (*σ*_*i*_||*S*) does not equal *D*_KL_ (*S*||*σ*_*i*_), we always applied it in the same direction, that is, *D*_KL_ (*σ*_*i*_||*S*).

To further validate our results, we repeated the analysis after applying a higher velocity threshold (10 cm s^−1^) as well as after the exclusion of HD cells with multiple receptive fields or noisy unit clusters. To detect multipeaked cells, multipeak score was computed for each cell, defined as the mean firing rate of a normalized tuning curve at the angular values outside of its primary peak (±width of the curve from the angle of its maximum firing rate). To identify noisy unit clusters, for each cell, a spike train auto-correlogram was computed at ±100 ms (1 ms bins) and the cluster contamination score was defined as the ratio between the value at 0.1 ms and the highest value. Noisy clusters were defined as those with cluster contamination scores above 0.2 (11% of all cells in the dataset).

### Isomap analysis of HD tuning curve auto-correlograms

Cross-validated auto-correlograms of each cell’s HD tuning curve were computed as described above. Then, nonlinear dimensionality reduction was performed using the Isomap algorithm^39^. The parameters were set to 12 nearest neighbors and two dimensions. When mapping the first three Fourier components onto the resulting embedding, we normalized their power to the total sum of their powers.

### Anatomical tract tracing

VGAT-IRES-Cre mice were injected with an adeno-associated virus vector (AAV 2/9 CAG-Flex-EGFP, titer: 4 to 5 × 10^12^ GC per ml; Neurophotonics; 500 μl per injection site) bilaterally into the TRN as described above. Four weeks after injections, animals were perfused transcradially with 4% PFA in phosphate-buffered saline and their brains were then cut coronally in 40 μm sections with a freezing microtome. The sections were counterstained with blue NeuroTrace (Thermo Fisher Scientific; 1:200 dilution) and mounted on slides with coronal z-stacks of the sections containing the rostral thalamus that were taken with a Leica SP-8 confocal microscope at ×10 magnification, using the same settings for all sections. GFP signal was acquired using the 473 nm excitation laser line. Z-projections of each stack were then obtained using ImageJ (version 1.52). Quantification of anterograde tracing was done in ImageJ. The images were converted to grayscale, and rectangular regions of interest (ROI) were defined within each thalamic nucleus. Average pixel intensity per ROI was then calculated using the ‘Measure’ function.

### Gain modulation analysis: experiment

Epochs of optogenetic stimulation (light ON) consisted of time periods when the laser was switched on (240 pulses of 1-s duration, 240 s per session). Control epochs (light OFF) were defined as time periods in between light pulses (240 periods of 4-s duration, 960 s per session). Light ON and light OFF tuning curves were computed from these periods. For analysis of tuning curve width, HD tuning curves were then computed in bins of 1° and smoothed with a Gaussian kernel of 3° s.d.

For analysis of additive and multiplicative gain, to preserve the independence of individual angular bins, HD tuning curves were instead computed in bins of 6° with no Gaussian smoothing applied. Light ON (that is, high gain) and light OFF (that is, baseline) tuning curves for each cell were then normalized by dividing them by the maximum value of the baseline tuning curve. To calculate the additive and multiplicative factors for each cell, a linear fit between high gain and baseline tuning curve vectors was then obtained using the MATLAB polyfit function. The slope of the resulting linear fit and its Y intercept were then taken as multiplicative and additive factors, respectively.

Because estimation of gain factors is not possible for cells that are not gain-modulated and/or their baseline and high gain tuning curves are not significantly correlated, we excluded cells in the ArchT group that did not show statistically significant modulation by light and did not show a statistically significant correlation between baseline and high gain tuning curves. Significant modulation by light was computed by, for each cell, calculating the average firing rate during each baseline and each high gain epoch, and subsequently comparing the two sets of values with a Wilcoxon signed-rank test with a significance threshold of 0.05. Significance of the tuning curve correlation was determined by the Pearson correlation coefficient (significance threshold of 0.05). This led to the exclusion of 1 of 47 PoSub-FS cells and no cells from other populations.

### Gain modulation analysis: simulations

The relationship between tuning curve correlations and additive/multiplicative factors was first explored in simple simulations involving monotonically increasing vectors (correlation coefficient (*r*) between vectors = 1). Noise was added separately to each vector by offsetting each point by a random value drawn from a Gaussian distribution. We varied the s.d. of the noise distribution to alter the value of the Pearson correlation coefficient between the two vectors. We then performed a linear regression between the two vectors using the MATLAB *polyfit* function and computed the additive and multiplicative factors as outlined above. The procedure was performed 1,000 times for each value of s.d. of the noise distribution. This enabled us to quantify how the apparent gain factor estimations change as a function of vector correlation.

On the basis of above simple simulations, it became apparent that our methods might have underestimated the amount of multiplicative gain in PoSub-FS cells. Consequently, we next sought to determine whether considerably low tuning curve correlations in the PoSub-FS cells preclude the detection of multiplicative gain via linear regression. To that end, we applied a method analogous to the one described above to real PoSub-FS cell tuning curves. We used the baseline tuning curves from the ArchT group and computed simulated high-gain tuning curves by applying Gaussian noise independently to each tuning curve until we obtained a similar distribution of tuning curve correlations to the one observed experimentally. We subsequently applied either multiplicative gain (multiplying each point on the tuning curve by 1.2) or additive gain (by adding 20% of the maximum tuning curve value to each point on the tuning curve) before applying Gaussian noise. These simulations allowed us to establish that multiplicative gain is indeed detectable in tuning curves with correlation values similar to those observed experimentally in PoSub-FS cells and that our experimental results in PoSub-FS cells are best explained by significant additive gain.

### Model fitting and theory

First, we derived network parameters based on the statistics of observed PoSub-FS cell tuning curves. Specifically, we characterized the connectivity from ADN- and PoSub-HD cells to PoSub-FS cells using mean and s.d. of connection weights. To differentiate the contributions of ADN and PoSub inputs, we optimized these parameters. The goal was to closely replicate the firing rate statistics and Fourier tuning profiles of the PoSub-FS cells. The optimization process is governed by the following three constraints, formulated as a loss function minimized through a gradient descent procedure: (1) matching the firing rates between simulated and recorded PoSub-FS cells, (2) equating the variance of the firing rates and (3) ensuring similarity in tuning curve shapes by aligning the variances of the actual Fourier coefficients.

Next, we theoretically derived the asymptotic behavior of the proportion of simulated cells with a certain Fourier power. We showed that this proportion becomes independent of the weight mean and s.d. in the limit of low or high variance relative to the mean.

Finally, we aimed to determine the actual distribution of output neuron tuning curves. We show that tuning curves of randomly connected linear neurons, with normally distributed weights *W*, are constrained to lie in the subspace spanned by the singular components of their input tuning curves. Furthermore, they are distributed in this subspace according to a multivariate normal distribution with diagonal covariance. As a result, Fourier components of an output tuning curve are independent of each other, as observed in the population of PoSub-FS cells.

Supplementary Methods provide further details of the model fitting procedure, the theoretical distribution of FS tuning curves and a generalization to other synaptic weight distributions.

### Data analysis and statistics

Analyses presented in Fig. 2 and Extended Data Fig. 5 were conducted using software custom-written in Python 3.9.7 with the following libraries: Scipy (version 1.7.3), Matplotlib (3.5.1), Numpy (1.20.3), Uncertainties (3.1.17) and Hdf5storage (0.1.18). All other analyses were conducted using MATLAB R2020b (Mathworks) with TStoolbox 2.0 and Circular Statistics Toolbox version 1.21 (ref. ^67^). Statistical comparisons were performed with nonparametric tests (Mann–Whitney *U* test, Wilcoxon signed-rank test) or analysis of variance (ANOVA) with multiple comparisons, where applicable. For ANOVA, data distribution was assumed to be normal but this was not formally tested. All statistical tests were two-tailed. No statistical methods were used to predetermine sample sizes, but our sample sizes are similar to those reported in previous publications^34,48,49^. Data collection and analysis were not performed blind to the conditions of the experiments. Data collection was not randomized as individual experiments were performed in sequence.

### Reporting summary

Further information on research design is available in the Nature Portfolio Reporting Summary linked to this article.

## Online content

Any methods, additional references, Nature Portfolio reporting summaries, source data, extended data, supplementary information, acknowledgements, peer review information; details of author contributions and competing interests; and statements of data and code availability are available at 10.1038/s41593-024-01588-5.

## Supplementary information

Supplementary Information Supplementary Methods.

Reporting Summary

## Data Availability

The datasets used in this study can be found at: 10.6084/m9.figshare.24921252.

## Code availability

The code generated is available at the following address: 10.6084/m9.figshare.24921252.
