# Supplementary material for: Local origin of excitatory–inhibitory tuning equivalence in a cortical network
Source: Nat Neurosci. 2024 Mar 15;27(4):782–92. doi: 10.1038/s41593-024-01588-5 (PMC11001581; doi:10.1038/s41593-024-01588-5)
Supplement: Supplementary file 1 — Supplementary Methods. [file 41593_2024_1588_MOESM1_ESM.pdf]

nature neuroscience

Article https://doi.org/10.1038/s41593-024-01588-5

Local origin of excitatory–inhibitory tuning equivalence in a cortical network

In the format provided by the authors and unedited

Supplementary Methods

Fourier spectrum simulations – Model fitting

We aim to derive network parameters based on the statistics of observed tuning curves in FS cells. We focus on the two main input populations to these cells: the ADN and PoSub-HD cells. We characterize the connectivity to FS cells from these populations using mean and standard deviations of connection weights. To differentiate the contributions of ADN and PoSub inputs, we optimize these parameters. The goal is to closely replicate the firing rate statistics and Fourier tuning profiles of the FS cells. This optimization process involves a comparison of the input profiles from ADN and PoSub to each FS cell, revealing that while ADN inputs to PoSub-FS are virtually independent of head-direction, PoSub-HD inputs are specifically tuned.

The optimization process is governed by three constraints, formulated as a loss function minimized through a gradient descent procedure: (1) Matching the firing rates between simulated and recorded FS cells, (2) Equating the variance of the firing rate, and (3) Ensuring similarity in tuning curve shapes by aligning the variances of the actual Fourier coefficients.

Tuning curves are discretized in m_θ points and this discretization is as fine as needed to preserve all the information of the tuning curves. The tuning curves of a population of output neurons are represented by a (n_y, m_θ) matrix Y. These neurons receive inputs from n_X neurons of tuning curves X connected to Y by a synaptic matrix W so that, in the linear case, $Y = WX$.

Let us derive the first two objectives on the mean and variance of output firing rates.

First, considering that PoSub-FS cells, represented by tuning curves Y, receive inputs from both PoSub-HD and ADN neurons, we have in the linear regime:

$$Y(\theta) = W_{AD}X_{AD}(\theta) + W_{pos}X_{pos}(\theta)$$

Taking the mean over the head-direction:

$$< Y >_\theta = W_{AD} < X_{AD} >_\theta + W_{pos} < X_{pos} >_\theta$$

Taking the variance over the head-direction:

$$V\left(Y_j\right)_\theta = \sum_{j=1}^{n_{ad}} w_{ad,ij}^2 V\left(X_{ad\,j}\right)_\theta + \sum_{j=1}^{n_{pos}} w_{pos,ij}^2 V\left(X_{pos\,j}\right)_\theta$$

Average across many simulations of one output cells:

$$E(< Y >_\theta) = \mu_{weff_{ad}} n_{AD} E(< X_{ad} >_\theta) + \mu_{weff_{pos}} n_{pos} E\left(< X_{pos} >_\theta\right)$$

$$E(V(Y)_\theta) = (\mu_{weff_{AD}}^2 + \sigma_{weff_{AD}}^2) n_{AD} E(V(X_{ad})_\theta) + (\mu_{weff_{pos}}^2 + \sigma_{weff_{pos}}^2) n_{pos} E\left(V\left(X_{pos}\right)_\theta\right)$$

which provides us with the first two losses, optimized:

$$L_{mean} = \left(E(< Y >_\theta) - \ \mu_{weff_{ad}} n_{AD} E(< X_{ad} >_\theta) + \mu_{weff_{pos}} n_{pos} E\left(< X_{pos} >_\theta\right)\right)^2$$

$$L_{variance} = \Big(E(V(Y)_\theta) - (\mu^2_{weff_{AD}} + \sigma^2_{weff_{AD}})n_{AD}E(V(X_{ad})_\theta) + \left(\mu^2_{weff_{pos}} + \sigma^2_{weff_{pos}}\right) n_{pos} E\left(V\left(X_{pos}\right)_\theta\right)\Big)^2$$

Next, we derive the third constraint on the Fourier coefficients:

$$Y = W_{ad}X_{ad} + W_{pos}X_{pos}$$

$$Y - E\big(Y(\theta)\big) = \big(W_{ad} - \mu_{W_{ad}}\mathbf{1}\big)X_{ad} + (W_{pos} - \mu_{W_{pos}}\mathbf{1})X_{pos}$$

The weights are independent and in the limit of a large number of simulations we have:

$$\frac{1}{n_y}\big(W_{ad} - \mu_{W_{ad}}\mathbf{1}\big)^T\big(W_{ad} - \mu_{W_{ad}}\mathbf{1}\big)_{ij} = \frac{1}{n_y}\sum_{k=1}^{n_y}(w_{ki} - \mu_k)\big(w_{kj} - \mu_k\big) \rightarrow \delta_{ij}\sigma_{w_{ad}}$$

$$\big(W_{ad} - \mu_{W_{ad}}\mathbf{1}\big)^T\left(W_{pos} - \mu_{W_{pos}}\mathbf{1}\right)_{ij} \rightarrow 0$$

Therefore:

$$\frac{1}{n_y}\big(Y - E\big(Y(\theta)\big)^T\big(Y - E\big(Y(\theta)\big) = \sigma^2_{w_{ad}}X^T_{ad}X_{ad} + \sigma^2_{w_{pos}}X^T_{pos}X_{pos}$$

This equation is then projected on the Fourier basis:

$$\frac{1}{n_y}(YV_F - E(YV_F)^T\big(YV_F - E(YV_F)\big) = \sigma^2_{w_{ad}}n_{ad}\frac{(V_F^TX^T_{ad}X_{ad}V_F)}{n_{ad}} + \frac{\sigma^2_{w_{pos}}n_{pos}\big(V_F^TX^T_{pos}X_{pos}V_F\big)}{n_{pos}}$$

Taking the diagonal of this equations, we obtain a system of equations linking the variance of the PoSub-FS cell Fourier coefficients with the variance of the PoSub- and ADN-HD cell Fourier coefficients.

As for the previous equation, we take the squared difference of the right hands side and left-hand side of these equations followed by their sum to obtain our third loss $L_{Fourier}$.

The three losses are then summed to produce the final loss. L_{mean} and $L_{variance}$ are scaled by 0.25 time the left-hand side of their original equation to balance the three losses. Optimization is run with *pytorch* and stochastic gradient descent, with optimizer Adam and learning rate 0.0001 for 200000 steps. To have a fair comparison between ADN and PoSub inputs, the number of ADN inputs was artificially expanded by a factor 10, matching the number of PoSub inputs. This was achieved by randomly shifting circularly true tuning curves before computing the statistics used in the loss computations.

Stochastic gradient descent was necessary to obtain a parallel optimization of the three objectives. Optimizing only $L_{Fourier}$ can be achieved analytically but would result in tuning curves of unreasonable mean or variance.

Fourier spectrum simulations – theory

We theoretically derive the asymptotic behavior of the proportion of simulated cells with a certain Fourier power. We show that this proportion becomes independent of the weights mean and standard deviation in the limit of low or high variance relative to the mean. This proportion is a statistic of the ratios of each Fourier power over the sum of all Fourier power. Consequently, it is sufficient to show that this ratio converges to a limit distribution independent of the weights mean and variance.

Here, we sought to determine the average response of neurons to an encoded feature θ (i.e. its tuning) as a function of the tuning of its inputs and their synaptic weights. At first, we assume that the response of the neuron y_j is linear and free of noise. In this case, the tuning curve is:

$y_j(\theta) = \sum_{i=1}^{n} w_{ji}\, x_i(\theta)$,

where x are the tuning curves of presynaptic neurons, θ is the feature, w_{ji} the synaptic weight from input neuron i to output neuron j, and *n* the number of input neurons.

Let us rewrite each weight as $w_{ji} = \mu_w + v_{ji}\sigma_w$

Where v_{ji} is the z-scored weight $v_{ji} = \frac{(w_{ji} - \mu_w)}{\sigma_w}$

For a set of n input tuning curves $x(\theta)$,

$$y_j(\theta) = \mu_w \sum_{i=1}^{n} x_i(\theta) + \sigma_w \sum_{i=1}^{n} v_{ji} x_i(\theta)$$

For normally distributed weights, the distribution of v_{ji} is independent of the mean and standard variation of the weight distribution. Consequently $\sum_{i=1}^{n} v_i x_i(\theta)$ is independent of the mean and standard deviation. Let us write the Fourier coefficients of this term as b_l, so that we have:

$\sum_{i=1}^{n} v_{ji}\, x_i(\theta) = \sum_{l=1}^{d} b_l\, e_l(\theta)$,

where *e* is the discrete Fourier basis:

$$e_{2l}(\theta) = \cos(l\theta)\,;\ e_{2l+1}(\theta) = \sin(l\theta)$$

Noting the Fourier coefficients of the sum of input tuning curves as a_l :

$$\sum_{i=1}^{n} x_i(\theta) = \sum_{l=1}^{d} a_l\, e_l(\theta)$$

the Fourier coefficients of the output tuning curves can be expressed as:

$$y_l = \sigma_w\, b_l + \mu_w\, a_l$$

Finally, the Fourier power of the output tuning curves are:

$$c_l = \sigma_w^2 \left(b_{2l}^2 + b_{2l+1}^2\right) + \sigma_w \mu_w (b_{2l} a_{2l} + b_{2l+1} a_{2l+1}) + \mu_w^2 \left(a_{2l}^2 + a_{(2l+1)}^2\right)$$

In the limit $\sigma_w \gg \mu_w$ or $\sigma_w \ll \mu_w$, the ratio of Fourier power r_l will only depend on the distribution of the b_l or of the a_l.

If $\sigma_w \gg \mu_w$, we have $r_l = \frac{c_l}{\sum_{l=1}^{\frac{d}{2}} c_l} = \frac{(b_{2l}^2 + b_{2l+1}^2)}{\sum_{l=1}^{d} b_l^2}$

And if $\sigma_w \ll \mu_w$ we have $r_l = \frac{(a_{2l}^2 + a_{2l+1}^2)}{\sum_{l=1}^{d} a_l^2}$

For normally distributed weights, b_l and a_l are independent of the weight mean and standard deviation (by definition). Therefore, the ratio of Fourier power converges to a distribution that is independent of the weight mean and standard deviation. By extension, the proportion of cells in the data showing maximum power in a certain fold (i.e. Fourier component) is independent of the weight statistics. In the case $\sigma_w \gg \mu_w$, the power ratio depends only on the spectrum of the weighted sum of input tuning curves.

Distribution of output cell tuning curves

We next demonstrate that the Fourier spectrum of the output tuning curves is entirely determined by the input tuning curve spectrum – independent of the synaptic weight mean and standard deviation, as well as the choice of weight distribution.

We show that in the limit of a large number of input cells, the Fourier coefficients of the outputs tuning curves are distributed as independent normal distributions, even for non-normally distributed weights, similar to PoSub-FS cells for which Fourier power is often concentrated in only one or two components.

We first present the case of a linear combination with normally distributed weights W (mean μ_W and standard variation σ_W) and then generalize it to other weights.

Demonstration for normally distributed weights

The exact distribution of tuning curves can be derived by projecting the input tuning curves in their natural basis i.e. by decomposing them with a singular value decomposition (SVD). The (n, m_θ) tuning curve matrix X of n neurons is expressed as $X = USV^T$.

To determine the statistics of the output tuning curve $y = WX$, one can project y onto the singular vectors of X . The projection of y onto the l-th singular vector is:

$$\widehat{y_l} = Yv_l^T = \sum_{i=1}^{n} w_i\, u_{il} s_l$$

It is sufficient to know the value of the first $d = rank(X)$ projection $\{\widehat{y_1}, \dots, \widehat{y_d}\}$ to fully reconstruct the tuning curve in the feature space: $\{y_1, \dots., y_{m_\theta}\}$. Indeed, the projection onto the $d+1, \dots, m_\theta$

singular vectors are null. Therefore, the distribution of tuning curves can be equivalently described either in feature space or singular component space.

This projection $\widehat{y_l}$ is an affine transformation of the random multivariate normal variable W by $US_{(:,1:d)}$. As $US_{(:,1:d)}$ forms an orthogonal basis, the $\widehat{y_l}$ are independent (up to $l = d$). As w_k are independent samples from a normal probability distribution, the random variable $\widehat{y_l}$ is itself distributed according to a normal distribution $N(\widehat{\mu_l}, \widehat{\sigma_l})$ where:

$$\widehat{\mu_l} = \mu_W s_l \sum_{i=1}^{n} u_{il}$$

$$\widehat{\sigma_l} = s_l \sigma_W$$

In summary, and quite intuitively, tuning curves of randomly connected linear neurons are constrained to lie in the subspace spanned by the singular components of their input tuning curves. They are distributed in this subspace according to a multivariate normal distribution with diagonal covariance. The weight standard deviation and l-th input singular value control the variance of $\widehat{y_l}$.

The above result was derived for normal distributions. For non-normally distributed weights (as it is the case of lognormally distributed positive weights), one potential approach would be to use the central limit theorem (CLT), on the sequence of random variable $w_1 u_{1l}, \dots, w_n u_{nl}$. However, the CLT assumes identically distributed random variables, which is not the case here because the u_{il} are not identically distributed.

Extension of the proof to non-normally distributed weights:

Here, we show that the main result holds anyway for non-normally distributed weights in the limit of large number of input excitatory cells n, under some realistic assumptions on the u_{il}. To this end, we use a generalized version of the CLT, the so-called Lyapunov Central limit theorem.

The Lyapunov central limit theorem is the following:

Suppose $V_1, \dots V_n$ are independent random variables with mean 0 and finite variance σ_i^2.

Let $v_n^2 = \sum_{i=1}^{n} \sigma_i^2$ denote the sum of their variance and $S_n = V_1 + \cdots + V_n$.

If for some $\delta > 0$ the so-called Lyapunov condition is verified:

$$\lim_{n\to\infty} \frac{1}{v_n^{2+\delta}} \sum_{i=1}^{n} E(|V_i|^{\delta+2}) = 0$$

Then $\lim\limits_{n\to\infty} \frac{S_n}{v_n} = \mathcal{N}(0,1)$ in distribution.

We will apply the theorem to each projection of the output tuning curves onto the right singular vectors of the input tuning curves. This will demonstrate the normality of each projection. We will then prove the independence of these projections.

Let l be the index of the right singular vector of the input tuning curves.

We note W_i the random variable describing the weights of the i-th input.

The dependence of the tuning curve left singular vector on the number of cells can be made explicit:

$$u_{il} = 1 + \epsilon_{il}(n)$$

For example, for a set of von Mises tuning curve (i.e., canonical HD tuning curves) with preferred direction uniformly spread on the circle, we have:

$$u_{il} = \cos\left(\frac{2\pi i}{n} l\right) = 1 + \sum_{p=1}^{\infty} \frac{(-1)^p \left(2\pi \frac{i}{n} l\right)^{2p}}{2p!}$$

The projection of an output tuning curves on the l-th singular vector is:

$$\hat{y}_l = \sum_{i=0}^{n} W_i s_l + \sum_{i=0}^{n} W_i s_l \epsilon_{il}(n)$$

The term $\sum_{i=0}^{n} W_i s_l$ is a sum of identically independently distributed weights and the CLT will apply.

For the second term $\sum_{i=0}^{n} W_i s_l \epsilon_{il}(n)$ we demonstrate that the Lyapunov condition holds under certain assumption for the $\epsilon_{il}(n)$.

We define the random variable $V_i = (W_i - \mu_w) s_l \epsilon_{il}(n)$ of mean 0 and finite.

First, we compute the variance of the sum:

$v_n^2 = s_l^2 \sigma_w^2 \sum_{i=1}^{n} \epsilon_{il}(n)^2$

We have

$$\sum_{i=1}^{n} \epsilon_{il}(n)^2 = \sum_{i=1}^{n} (u_{il} - 1)^2 = n - 2\left(\sum_{i=1}^{n} u_{il}\right) + \sum_{i=1}^{n} u_{il}^2$$

$$\sum_{i=1}^{n} \epsilon_{il}(n)^2 = n - 2\left(\sum_{i=1}^{n} u_{il}\right) + 1$$

<u>Assumption 1:</u> $\sum_{i=1}^{n} u_{il}$ is finite when n grows to infinity, i.e $\sum_{i=1}^{n} u_{il} = O(1)$

Note: this is the case for the set of perfect von Mises tuning curves, as $\sum_{i=1}^{n} u_{il} \to_{n\to\infty} 0$

Under assumption 1:

$$\sum_{i=1}^{n} \epsilon_{il}(n)^2 = n + O(1).$$

$$v_n^2 = s_l^2 \sigma_w^2 \sum_{i=1}^{n} \epsilon_{il}(n)^2 = s_l^2 \sigma_w^2 (n + O(1))$$

$$v_n = s_l \sigma_w \sqrt{n + O(1)}$$

Next let us compute the sum of expectation term. For every $i \in [1, n]$

$$E\left(\left((W_i - \mu_w) s_l \epsilon_{il}(n)\right)^{2+\delta}\right) = (s_l \epsilon_{il}(n))^{2+\delta} E\left((W_i - \mu_w)^{2+\delta}\right)$$

The term $E\left((W_i - \mu_w)^{2+\delta}\right)$ is a statistic of the weights, independent of the random variable (i.e., independent of the index *i*).

$$E\left((W_0 - \mu_w)^{2+\delta}\right) = \cdots = E\left((W_n - \mu_w)^{2+\delta}\right)$$

Consequently

$$\frac{1}{v_n^{2+\delta}} \sum_{i=1}^{n} E\left(|V_i|^{\delta+2}\right) = \frac{E\left(\left(W_0 - \mu_w\right)^{2+\delta}\right) s_l^{2+\delta}}{\left(\sigma_w s_l \sqrt{n + O(1)}\right)^{2+\delta}} \sum_{i=1}^{n} \epsilon_{il}(n)^{2+\delta}$$

$$\frac{1}{v_n^{2+\delta}} \sum_{i=1}^{n} E\left(|V_i|^{\delta+2}\right) = \frac{E\left(\left(W_0 - \mu_w\right)^{2+\delta}\right)}{\sigma_w^{2+\delta}} \left(\frac{1}{\sqrt{n + O(1)}}\right)^{2+\delta} \sum_{i=1}^{n} \epsilon_{il}(n)^{2+\delta}$$

<u>Assumption 2:</u> $\left(\frac{1}{\sqrt{n+O(1)}}\right)^{2+\delta} \sum_{i=1}^{n} \epsilon_{il}(n)^{2+\delta} \rightarrow_{n\to\infty} 0$ i.e $\sum_{i=1}^{n} \epsilon_{il}(n)^{2+\delta} = o\left(n^{1+\frac{\delta}{2}}\right)$

Note: Let us show that assumption 2 holds for von Mises tuning curve:

$\epsilon_{il}(n) = 1 - \cos\left(\frac{2\pi i}{n} l\right)$

The sum $\frac{1}{n}\sum_{i=1}^{n} \epsilon_{il}(n)^{2+\delta}$ is in that case a Riemann sum that converges to the following integral:

$\frac{1}{n}\sum_{i=1}^{n} \epsilon_{il}(n)^{2+\delta} \rightarrow_{n\to\infty} \int_0^1 (1 - \cos(2\pi t))^{2+\delta} dt$ which is bounded

And therefore:

$$\left(\frac{1}{\sqrt{n+O(1)}}\right)^{2+\delta} \sum_{i=1}^{n} \epsilon_{il}(n)^{2+\delta} \sim \frac{n}{n^{1+\frac{\delta}{2}}} \frac{1}{n} \sum_{i=1}^{n} \epsilon_{il}(n)^{2+\delta} \sim \frac{cste}{n^{\frac{\delta}{2}}} \rightarrow_{n\to\infty} 0$$

Under the assumption 2, which we proved valid for a theoretical model of perfect input tuning curves, we have consequently:

$$\frac{1}{v_n^{2+\delta}}\sum_{i=1}^{n} E\left(|V_i|^{\delta+2}\right) \to_{n\to\infty} 0$$

This demonstrates the Lyapunov condition for the *l*-th projection. This proves that $\left(\frac{1}{s_l\sigma_w\sqrt{n+O(1)}}\right)\sum_{i=0}^{n} W_i s_l \epsilon_{il}(n)$ are normally distributed in the limit of large n, and consequently:

$\sum_{i=0}^{n} W_i s_l \epsilon_{il}(n)$ is also normally distributed in the limit of large n.

Therefore we obtained that both $\sum_{i=0}^{n} W_i s_l$ and $\sum_{i=0}^{n} W_i s_l \epsilon_{il}(n)$ are normally distributed, this proves that $\widehat{y_l} = \sum_{i=0}^{n} W_i s_l + \sum_{i=0}^{n} W_i s_l \epsilon_{il}(n)$ is also normally distributed.

We can compute the mean and variance of this normal distribution:

$$E(\hat{y}_l) = \sum_{i=1}^{n} E(W_i) s_l u_{il} = \mu_w s_l \sum_{i=1}^{n} u_{il}$$

$$Var(\hat{y}_l) = \sigma_w^2 s_l^2 \sum_{i=1}^{n} u_{il}^2 = \sigma_w^2 s_l^2$$

These statistics are true for all values of *n*, but the distribution itself converges to a normal distribution only at large *n*. The expectation and the variance of the limit distribution will be the limit of the expectation and variance computed above.

The limit statistics are:

$$\hat{\mu}_l = \mu_w s_l \sum_{i=1}^{\infty} u_{il}$$

$$\hat{\sigma}_l = \sigma_w s_l$$

Finally, the independence of the different projections simply stems from the fact that the covariance matrix is diagonal, as this property is the same as for the normally distributed weights. Therefore, $\widehat{y_1}, \dots, \widehat{y_d}$ are indeed independent. The output tuning curve projection on the right singular vector of the tuning curves are thus normally distributed and independent.

For canonical tuning curves, modeled as von Mises' distribution, the singular vectors are exactly the Fourier basis up to a proportionality coefficient. Here, we thus have demonstrated that, under realistic assumptions, the Fourier components of output tuning curves are independent and normally distributed with a variance depending on the weight variance. This is exactly what is observed in the data: while all PoSub-HD cells have qualitatively the same Fourier spectrum, each PoSub-FS cell has a unique spectrum, many of which concentrating power in only one or two components.
