# Supplementary material for: Local origin of excitatory–inhibitory tuning equivalence in a cortical network
Source: Nat Neurosci. 2024 Mar 15;27(4):782–92. doi: 10.1038/s41593-024-01588-5 (PMC11001581; doi:10.1038/s41593-024-01588-5)
Supplement: Supplementary file 2 — Reporting Summary [file 41593_2024_1588_MOESM2_ESM.pdf]

nature portfolio

Corresponding author(s): Adrien Peyrache

Last updated by author(s): Jan 2, 2024

Reporting Summary

Nature Portfolio wishes to improve the reproducibility of the work that we publish. This form provides structure for consistency and transparency in reporting. For further information on Nature Portfolio policies, see our Editorial Policies and the Editorial Policy Checklist.

Statistics

For all statistical analyses, confirm that the following items are present in the figure legend, table legend, main text, or Methods section.

n/a	Confirmed	
☐	☒	The exact sample size (*n*) for each experimental group/condition, given as a discrete number and unit of measurement
☐	☒	A statement on whether measurements were taken from distinct samples or whether the same sample was measured repeatedly
☐	☒	The statistical test(s) used AND whether they are one- or two-sided<br>*Only common tests should be described solely by name; describe more complex techniques in the Methods section.*
☒	☐	A description of all covariates tested
☐	☒	A description of any assumptions or corrections, such as tests of normality and adjustment for multiple comparisons
☐	☒	A full description of the statistical parameters including central tendency (e.g. means) or other basic estimates (e.g. regression coefficient) AND variation (e.g. standard deviation) or associated estimates of uncertainty (e.g. confidence intervals)
☐	☒	For null hypothesis testing, the test statistic (e.g. *F*, *t*, *r*) with confidence intervals, effect sizes, degrees of freedom and *P* value noted<br>*Give P values as exact values whenever suitable.*
☒	☐	For Bayesian analysis, information on the choice of priors and Markov chain Monte Carlo settings
☒	☐	For hierarchical and complex designs, identification of the appropriate level for tests and full reporting of outcomes
☐	☒	Estimates of effect sizes (e.g. Cohen's *d*, Pearson's *r*), indicating how they were calculated

Our web collection on statistics for biologists contains articles on many of the points above.

Software and code

Policy information about availability of computer code

Data collection	Commercial software:<br>Motive (Optitrack) version 2.0<br>Intan RHX (Intan Technologies) version 3.0<br><br>Open-source software:<br>SpikeGLX version 3.0<br>https://billkarsh.github.io/SpikeGLX/
Data analysis	Commercial software:<br>MATLAB (MathWorks) version R2020b<br><br>Open-source software:<br>Klusters version 2.0<br>ImageJ version 1.52<br>Python 3.9.7<br><br>MATLAB open-source packages:<br>TStoolbox version 2.0<br>https://github.com/PeyracheLab/TStoolbox<br>Kilosort version 2.0<br>https://github.com/MouseLand/Kilosort/releases/tag/v2.0

Circular Statistics Toolbox version 1.21
https://www.mathworks.com/matlabcentral/fileexchange/10676-circular-statistics-toolbox-directional-statistics
Matlab Toolbox for Dimensionality Reduction version 0.8.1b
https://lvdmaaten.github.io/drtoolbox/
SleepScoreMaster version downloaded in January 2021
https://github.com/buzsakilab/b
uzcode/tree/master/detectors/detectStates/SleepScoreMaster
TheStateEditor version downloaded in January 2021
https://github.com/buzsakilab/TheStateEditor
Custom code written for MATLAB by A.J.D. and A.P.

Python open-source packages:
Scipy version 1.7.3
https://scipy.org/
Matplotlib version 3.5.1
https://matplotlib.org/
hdf5storage version 0.1.18
https://pypi.org/project/hdf5storage/
numpy version 1.20.3
https://numpy.org/
uncertainties version 3.1.17
Pytorch 1.12.1
https://pytorch.org/
The code used to perform analyses in the manuscript can be found at: https://doi.org/10.6084/m9.figshare.24921252

For manuscripts utilizing custom algorithms or software that are central to the research but not yet described in published literature, software must be made available to editors and reviewers. We strongly encourage code deposition in a community repository (e.g. GitHub). See the Nature Portfolio guidelines for submitting code & software for further information.

Data

Policy information about availability of data

All manuscripts must include a data availability statement. This statement should provide the following information, where applicable:

- Accession codes, unique identifiers, or web links for publicly available datasets
- A description of any restrictions on data availability
- For clinical datasets or third party data, please ensure that the statement adheres to our policy

The datasets used in this study can be found at: https://doi.org/10.6084/m9.figshare.24921252

Research involving human participants, their data, or biological material

Policy information about studies with human participants or human data. See also policy information about sex, gender (identity/presentation), and sexual orientation and race, ethnicity and racism.

Reporting on sex and gender	N/A
Reporting on race, ethnicity, or other socially relevant groupings	N/A
Population characteristics	N/A
Recruitment	N/A
Ethics oversight	N/A

Note that full information on the approval of the study protocol must also be provided in the manuscript.

Field-specific reporting

Please select the one below that is the best fit for your research. If you are not sure, read the appropriate sections before making your selection.

☒ Life sciences ☐ Behavioural & social sciences ☐ Ecological, evolutionary & environmental sciences

For a reference copy of the document with all sections, see nature.com/documents/nr-reporting-summary-flat.pdf

Life sciences study design

All studies must disclose on these points even when the disclosure is negative.

Sample size	No sample size calculations were performed. For mice implanted in postsubiculum, data from two separate experiments (vertical implantation

Sample size	across cortical layers, angled implantation along cortical layers) was pooled, resulting in a large cohort of 32 mice and nearly 3000 single units. We did not observe any substantial differences in results across the two implant configurations. For mice implanted in the anterodorsal thalamic nucleus the total number of animals (n = 8) and number of single units (n = 228) due to the small size of this brain structure and the high targeting difficulty associated with it.<br><br>Samples included all available cells that matched the classification criteria for the relevant cell type. We reasoned that due to the high heterogeneity of tuning in PoSub-FS compared to HD cells it was critical to direct our resources to maximise the sample size of this cell group. The total number of FS cells in our dataset (n = 427) is high in comparison with other studies of cortical interneuron tuning. Since HD cells show much more homogenous tuning, we judge the sample size of 97 ADN-HD cells to be sufficient to quantify the tuning properties of these neurons. This sample size is comparable to, and often exceeds, those reported in other papers about ADN-HD cells.<br><br>In optogenetic experiments, the responses cells to the optogenetic manipulation were relatively homogenous within each cell group, which in our opinion justifies the smaller sample size.
Data exclusions	For the main analysis, the only exclusion criterion was off-target implantation, as judged by absence of sharply-tuned HD cells and histological assessment. For the analysis of tuning stability across environments, only mice that underwent the additional exploration session in a triangular environment (n = 20) were included. For the cue rotation analysis, only the mice that underwent the cue rotation experiment and in which a substantial effect of cue rotation on receptive fields was observed were included (n = 6). Mice which underwent the cue rotation protocol but in which HD cells did not remap following cue rotation (n = 5) were excluded. All the above criteria were pre-established.<br><br>For REM sleep analysis, only mice in which at least 2 minutes of REM sleep was recorded (n = 28) were included, and those with less than 2 min of REM sleep (n = 6) were excluded. We observed that less than 2 min of REM sleep is not enough to reliably calculate the cross-correlations between cell pairs or perform the manifold analysis. This criterion was not pre-established.<br><br>For the purpose of comparison with simulated data (Extended Data Figure 5) HD information for the recorded population of FS cells was calculated after exclusion of two outliers (out of 427 cells). Both outliers had narrow waveforms and high firing rates consistent with putative FS cells, but their tuning curves were indistinguishable from canonical HD cells, which resulted in HD information scores orders of magnitude higher than other putative FS cells (see Figure 1f).
Replication	For PoSub recordings, results from mice implanted vertically (n = 14) were reproduced in the next cohort of mice implanted parallel to cortical layers (n = 18). All other experiments were conducted as single cohorts and replication was not attempted.
Randomization	Allocation of animals to either ADN or PoSub implant group was not random as these experiments were carried out in sequence and over a period of 3 years. For optogenetic experiments, animals were randomly allocated to either ArchT or control group.
Blinding	Experimenters were not blinded to the group allocation during data collection or analysis since group allocation was easily deducible due to different appearance of the probe implant, obvious differences in neural activity patterns between PoSub and ADN and strong effects of the optogenetic manipulation on neural activity.

Reporting for specific materials, systems and methods

We require information from authors about some types of materials, experimental systems and methods used in many studies. Here, indicate whether each material, system or method listed is relevant to your study. If you are not sure if a list item applies to your research, read the appropriate section before selecting a response.

Materials & experimental systems

n/a	Involved in the study
☐	☒ Antibodies
☒	☐ Eukaryotic cell lines
☒	☐ Palaeontology and archaeology
☐	☒ Animals and other organisms
☒	☐ Clinical data
☒	☐ Dual use research of concern
☒	☐ Plants

Methods

n/a	Involved in the study
☒	☐ ChIP-seq
☒	☐ Flow cytometry
☒	☐ MRI-based neuroimaging

Antibodies

Antibodies used	Donkey anti-mouse secondary antibody for visualizing the probe tracts (Cy3, Cedarlane: 715-165-150, various batches)
Validation	No primary antibodies were used

Animals and other research organisms

Policy information about studies involving animals; ARRIVE guidelines recommended for reporting animal research, and Sex and Gender in Research

Laboratory animals	The subjects were adult (> 8 week old) male mice bred by crossing wild-type females on C57BL/6J background (Jackson laboratories

Laboratory animals	000664) with either homozygous male VGAT-IRES-Cre mice (Jackson laboratories 028862, n = 41) or PV-IRES-Cre mice (Jackson laboratories 017320, n = 3). An additional mouse (n = 1) implanted with a Neuropixel probe (Figure 1a-c) was a cross-bred C57BL/6J and FVB (Jackson laboratory 001800).
Wild animals	No wild animals were used
Reporting on sex	All experiments were conducted on male mice. Only male mice were used because female mice are smaller in size and thus unable to comfortably carry the microdrive implants used in the study.
Field-collected samples	No field-collected samples were used
Ethics oversight	All procedures were approved by the Animal Care Committee of the Montreal Neurological Institute at McGill University in accordance with Canadian Council on Animal Care guidelines.

Note that full information on the approval of the study protocol must also be provided in the manuscript.
